# A Review of Traditional Chinese Medicine Formulations and Natural Active Ingredients with Therapeutic Potential for Male Infertility Targeting Oxidative Stress

**DOI:** 10.3390/ph19010012

**Published:** 2025-12-20

**Authors:** Zhen Peng, Ning Zhang, Fengting Yin, Ling Kong, Hui Sun, Chang Liu, Yaning Wu, Chenyue Wang, Xijun Wang

**Affiliations:** 1 State Key Laboratory of Integration and Innovation of Classic Formula and Modern Chinese Medicine, National Chinmedomics Research Center, National TCM Key Laboratory of Serum Pharmacochemistry, Metabolomics Laboratory, Department of Pharmaceutical Analysis, Heilongjiang University of Chinese Medicine, Heping Road 24, Harbin 150040, China; pengzhenycps@163.com (Z.P.); zhangning@hljucm.net (N.Z.); yfengting2025@163.com (F.Y.); 15244624557@163.com (L.K.); lc_work_lc@163.com (C.L.); 18839387651@163.com (Y.W.); wcy04042023@163.com (C.W.); 2 State Key Laboratory of Quality Research in Chinese Medicine, Macau University of Science and Technology, Avenida Wai Long, Taipa, Macau

**Keywords:** traditional Chinese medicine compound, natural active ingredients, male reproductive function, oxidative stress, molecular mechanism

## Abstract

Male infertility has emerged as a significant global concern, with male factors accounting for approximately 30% to 50% of infertility cases. Oxidative stress is recognized as the primary pathological mechanism affecting sperm structure and function. The development and application of chemically synthesized drug therapies are limited by lengthy research and development processes and significant adverse effects. Conversely, Traditional Chinese Medicine (TCM) compounds offer promising clinical applications for enhancing male reproductive function, attributed to their distinctive advantages of multi-target coordination and holistic regulation. This paper systematically reviews classical TCM compounds, such as those that tonify the kidney and benefit essence, warm and invigorate kidney Yang, and replenish Qi and nourish blood. It also examines the molecular mechanisms of active natural ingredients, including flavonoids, polyphenols, terpenes, alkaloids, and polysaccharides. These compounds improve male fertility by modulating oxidative stress-related signaling pathways. Furthermore, this review anticipates future research trajectories and potential applications within this domain, with the objective of establishing a theoretical basis for the clinical treatment of idiopathic male infertility and the development of novel pharmacological interventions.

## 1. Introduction

### 1.1. The Global Burden of Male Infertility and Its Underlying Etiology

Infertility has become a significant global public health issue, with male factors estimated to contribute to approximately 30% to 50% of infertility cases, affecting about 8% to 12% of couples of reproductive age worldwide [1,2,3]. Existing studies indicate that male infertility is directly associated with a reduction in sperm quantity [4], decreased in motility [5,6], and morphological abnormalities [7]. Oxidative stress is identified as a critical factor contributing to the structural and functional abnormalities of sperm. The physiological concentration of reactive oxygen species (ROS) plays a regulatory role in sperm capacitation and the acrosome reaction by activating the tyrosine phosphorylation pathway at appropriate times [8,9]. In contrast to somatic cells, human sperm exhibit distinct structural features, including highly condensed chromatin and minimal cytoplasmic content, which contribute to a reduced presence of antioxidant enzymes such as superoxide dismutase and glutathione peroxidase. Additionally, the sperm cell membrane is enriched with unsaturated fatty acids, rendering it vulnerable to oxidative stress-induced damage [10,11,12,13].

**Figure 1:**
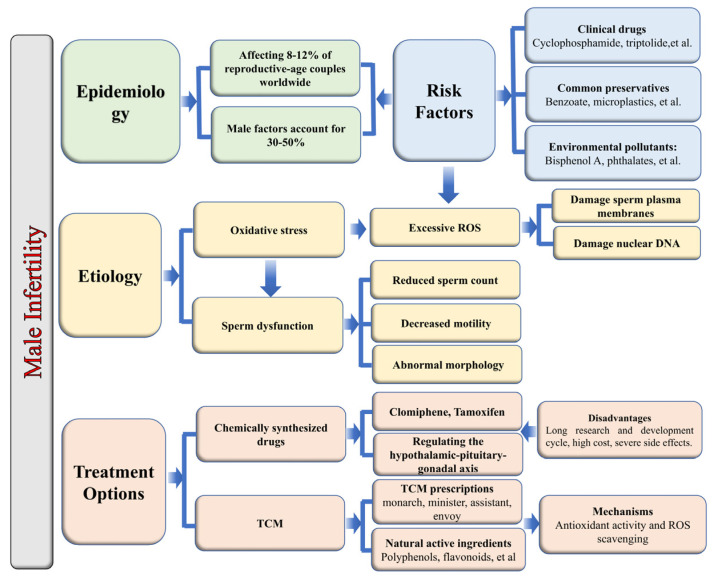
Epidemiology, etiology, risk factors, and treatment options for male infertility.

Exogenous factors, such as widely used clinical drugs like cyclophosphamide and triptolide, common preservatives such as benzoate, microplastics (specifically polystyrene microplastics), and various environmental pollutants (including bisphenol A, phthalates, aluminum, lead, and cadmium), can lead to an excessive accumulation of ROS in male mammals, particularly in human sperm. This accumulation disrupts redox homeostasis, impair sperm membrane function, and compromises the structural integrity of nuclear DNA, ultimately impairing male fertility [14,15,16,17,18]. Consequently, maintaining intracellular redox homeostasis in sperm is essential for preserving their normal physiological functions (Figure 1).

### 1.2. The Male Infertility Treatment Landscape and the Therapeutic Potential of TCM (Traditional Chinese Medicine)

The advancement of innovative synthetic pharmaceuticals for addressing male infertility is markedly hindered by a series of interrelated obstacles. Firstly, the research and development trajectory is both extended and financially demanding, largely attributable to the inherent complexity and prolonged timeline of human spermatogenesis. This necessitates extensive clinical trials characterized by high attrition rates. Secondly, pinpointing specific, druggable targets within the intricately coordinated process of germ cell development poses significant difficulties, compounded by the presence of the protective blood-testis barrier, which further impedes effective systemic drug delivery. Thirdly, the substantial etiological diversity inherent in male infertility complicates patient stratification and poses significant challenges in demonstrating consistent drug efficacy across clinical trials [19].

As a result, contemporary clinical management predominantly relies on hormonal interventions, such as selective estrogen receptor modulators like clomiphene and tamoxifen, which exhibit significant limitations. These agents function by stimulating gonadotropin secretion through the inhibition of hypothalamic estrogen feedback, rendering them effective only in patients with an intact hypothalamic-pituitary-gonadal axis, and largely ineffective in cases of primary testicular failure. Moreover, their systemic anti-estrogenic effects are associated with a spectrum of adverse outcomes, including vasomotor symptoms, mood disturbances, and an increased risk of thromboembolic events. Consequently, current therapeutic approaches are largely empirical, non-curative, and constrained by side effects, highlighting the pressing need for the development of novel therapeutic strategies that are specifically targeted towards the underlying pathophysiology [20,21,22].

In contrast, TCM has sustained a prominent role in addressing male infertility attributed to its unique advantages in holistic regulation and multi-target intervention [23]. Central to TCM is the application of herbal compounds, which achieve synergistic effects through the compatibility principle of “monarch, minister, assistant, and envoy”. This approach enhances overall male reproductive function and has demonstrated clear clinical efficacy [24,25,26]. A substantial body of research has shown that the polyphenols (particularly flavonoids), polysaccharides, and other natural active constituents found in TCM compounds possess significant antioxidant properties, effectively scavenging reactive oxygen species (ROS) and mitigating oxidative stress-induced damage [27,28]. Consequently, a comprehensive elucidation of the mechanisms by which TCM compounds enhance male reproductive function through the modulation of oxidative stress-related signaling pathways not only elucidates their pharmacological foundations but also provides a crucial theoretical basis for further research and clinical application in this field.

## 2. Traditional Chinese Medicine for Male Infertility

TCM prescriptions serve as vehicles for natural active ingredients. The foundational principle of “monarch, minister, assistant, and envoy” is designed to optimize the synergistic combination of active ingredients from diverse sources with varying pharmacological effects, thereby achieving the dual objectives of reducing toxicity and enhancing therapeutic efficacy. This study focuses on three classical Chinese herbal formulations commonly utilized in the treatment of male infertility. It reviews their representative prescriptions, the composition of TCM and the effects and mechanisms by which they improve male reproductive function (Table 1).

### 2.1. Prescriptions for “Tonifying Kidney and Benefiting Essence”: Herbal Strategies Targeting Male Reproductive Function

The category of TCM known as “nourishing the kidney and enhancing essence” constitutes a primary therapeutic strategy for addressing male infertility. This approach is grounded in the theoretical premise that “the kidney stores essence and governs reproduction”. Consequently, these formulations aim to enhance male reproductive function by strengthening kidney essence and maintaining the balance of Yin and Yang.

#### 2.1.1. Wuzi Yanzong Prescription

The Wuzi Yanzong Prescription (WZYZP), which originates from a distinguished Ming Dynasty formula, comprises five primary herbal ingredients: Lycii Fructus (*Lycium barbarum* L.), Cuscuta Semen (*Cuscuta chinensis* Lam.), Schisandrae Chinensis Fructus (*Schisandra chinensis* (Turcz.) Baill.), Rubi Fructus (*Rubus chingii* Hu), and Plantaginis Semen (*Plantago asiatica* L.). This prescription serves as a quintessential example of Chinese medicine that has been employed in the clinical treatment of male infertility for several centuries [42]. Within the context of TCM, Lycii Fructus and Cuscuta Semen are regarded as sovereign herbs, recognized for their capacity to nourish the liver and kidneys, thereby benefiting essence and blood. Schisandrae Chinensis Fructus and Rubi Fructus serve as ministerial herbs, further reinforcing the kidneys and essence. Plantaginis Semen acts as an adjuvant, characterized by its cooling and purgative properties. The prescription comprehensively addresses both kidney Yin and kidney Yang, functioning to tonify the kidneys and enhance essence.

With advancements in modern research methodologies, the active ingredients within the WZYZP have been progressively elucidated. Tan et al. employed ultra-performance liquid chromatography-electrospray ionization-linear ion trap-Orbitrap mass spectrometry to identify 106 compounds within the prescription, including flavonoids, alkaloids, terpenes, and other structural types. This analysis provides a substantial theoretical foundation for elucidating the prescription’s mechanism of action [43]. Currently, the bioactive constituents of specific medicinal ingredients, such as flavonoids in Cuscutae Semen and Plantaginis Semen, and polysaccharides in *Lycium barbarum* and Rubi Fructus, have been extensively investigated for their antioxidant, anti-inflammatory, and germ cell function-enhancing properties [44]. Previous research has demonstrated that WZYZP can significantly ameliorate the adverse effects of exogenous factors, such as ionizing radiation and hydrogen peroxide, on male reproductive function.

Testicular tissue is highly sensitive to ionizing radiation, and exposure can lead to spermatogenesis dysfunction, potentially resulting in temporary or permanent infertility [45]. In animal models, male mice exposed to pelvic X-ray irradiation were administered WZYZP (1 g/kg) via gavage daily, starting on the second day post-irradiation for a duration of three weeks. This intervention significantly mitigated X-ray-induced reductions in testicular weight, sperm count, and motility, and effectively reversed the decline in serum testosterone levels. Furthermore, WZYZP markedly decreased the levels of malondialdehyde (MDA) and the oxidative stress index (OSI) in testicular tissue while simultaneously enhancing the expression of proliferating cell nuclear antigen (PCNA). These findings suggest that WZYZP may offer protective effects against radiation-induced male reproductive damage through its dual mechanisms of antioxidant activity and promotion of cell proliferation [29].

In vitro cellular experiments further demonstrated that WZYZP elicited a dose-dependent reduction in oxidative damage and apoptosis in mouse Sertoli cells (TM4 cells) induced by hydrogen peroxide (H_2_O_2_). Notably, there was a significant enhancement in superoxide dismutase (SOD) activity and cell survival rates, accompanied by a marked decrease in MDA content, cell apoptosis rates, and mRNA expression levels of Caspase-3 [30].

Collectively, these findings from both in vivo and in vitro studies, suggest that WZYZP effectively mitigates oxidative stress responses induced by exogenous factors, enhances the functional state of spermatogenic and Sertoli cells, preserves the integrity of testicular tissue structure and physiological function, and ultimately contributes to the improvement of sperm quality [46,47].

#### 2.1.2. Bazi Bushen Prescription

The Bazi Bushen Prescription (BZBSP) is a modern TCM formulation derived from the classical WZYZP. This formulation includes ingredients such as Cuscutae Semen (*Cuscuta chinensis* Lam.), Lycii Fructus (*Lycium barbarum* L.), Schisandrae Chinensis Fructus (*Schisandra chinensis* (Turcz.) Baill.), Rubi Fructus (*Rubus chingii* Hu), Cnidii Fructus (*Cnidium monnieri* (L.) Cuss.), Rosae Laevigatae Fructus (*Rosa laevigata* Michx.), Semen Allii Tuberosi (*Allium tuberosum* Rottler ex Spreng.), Toosendan Fructus (*Melia toosendan* Sieb. et Zucc.), Epimedii Folium (*Epimedium brevicornu* Maxim.), Morindae Officinalis Radix (*Morinda officinalis* How), Cistanches Herba (*Cistanche deserticola* Y.C.Ma), Rehmanniae Radix Praeparata (Rehmannia glutinosa (Gaertn.) DC.), Cyathulae Radix (*Cyathula officinalis* Kuan), Ginseng Radix et Rhizoma (*Panax ginseng* C.A.Mey.), Cervi Cornu Pantotrichum (*Cervus nippon* Temminck) and Hippocampus (*Hippocampus kelloggi* Jordan et Snyder). Within the framework of TCM, this formulation is purported to nourish kidney essence and delay the aging process. It is primarily utilized to alleviate symptoms such as lumbar and knee soreness, dizziness, tinnitus, mental fatigue, and general weakness. Studies have identified that the prescription contains various natural antioxidant components, including hyperoside, chlorogenic acid, and icariin, which contribute to its capacity to eliminate ROS and mitigate cellular oxidative damage [48,49,50,51,52,53,54].

Aging is recognized as both a physiological and pathological process, marked by the progressive decline of multiple systems and organ functions [55,56]. The testis, a central organ of the male reproductive system, exhibits aging through diminished steroid hormone synthesis, impaired spermatogenesis, and reduced sperm quality [57,58]. Studies have indicated that oxidative stress is a fundamental mechanism underlying testicular aging with advancing age, the excessive accumulation of ROS in testicular tissue can induce mutations in sperm mitochondrial DNA and impair the steroidogenic function of interstitial cells, ultimately resulting in spermatogenesis disorders [58,59].

Lin et al. have provided crucial in vivo experimental evidence supporting the anti-aging effects of BZBSP on testicular function. In their study, a rapid aging mouse model was induced using D-galactose (D-gal) and sodium nitrite to systematically evaluate the intervention effects of BZBSP. The study’s findings demonstrated that the prescription markedly improved sperm quality in aging mice, as evidenced by improvements in sperm density and motility, along with the optimization of several kinematic parameters, including average path velocity, curvilinear velocity, lateral displacement amplitude, straightness, and linearity. Additionally, it effectively mitigated degenerative lesions in testicular tissue. At the mechanistic level, the Bazi Bushen formula was shown to significantly elevate the serum total antioxidant capacity (TAC) and the glutathione redox ratio [glutathione (GSH)/oxidized glutathione (GSSG)], while concurrently reducing serum levels of MDA and 8-hydroxydeoxyguanosine (8-OH-dG). These alterations collectively contributed to the restoration of redox homeostasis and the deceleration of testicular aging processes [31].

#### 2.1.3. Zuogui Pill

The Zuogui Pill (ZGP), originating from the “Jingyue Quanshu”, comprises several TCM ingredients, including Rehmanniae Radix Praeparata (*Rehmannia glutinosa* (Gaertn.) DC.), Corni Fructus (*Cornus officinalis* Sieb. et Zucc.), Dioscoreae Rhizoma (*Dioscorea opposita* Thunb.), Cervi Cornus Colla (*Cervus nippon* Temminck), Testudinis Carapacis et Plastri Colla (*Chinemys reevesii* (Gray)), Lycii Fructus (*Lycium barbarum* L.), Cuscutae Semen (*Cuscuta chinensis* Lam.) and Achyranthis Bidentatae Radix (*Achyranthes bidentata* Bl.) [60]. It serves as a quintessential prescription for “nourishing Yin and tonifying the kidney” within TCM. The pill is reputed for its capacity to nourish Yin, tonify the kidney, and replenish lean marrow. Currently, it is extensively utilized in the treatment of various conditions associated with true Yin deficiency, including disorders of the reproductive, skeletal, and nervous systems. Contemporary pharmacological research has elucidated that the therapeutic efficacy of ZGP is intricately linked to its diverse active constituents, such as iridoids, flavonoids, polysaccharides, and alkaloids, which collectively constitute the material foundation for its clinical effectiveness [61].

Within the field of reproductive health, ZGP not only promotes the proliferation and differentiation of spermatogonia but also exerts a systemic regulatory influence on male fertility by modulating the hypothalamic-pituitary-gonadal axis, thereby exhibiting characteristics of multi-target and holistic regulation [62]. The distinctive “cross-generational protection” effect of ZGP is particularly noteworthy. Lei et al. conducted a systematic evaluation of the long-term impact of ZGP on the reproductive function of male offspring rats by developing a rat model subjected to stress during pregnancy. In this study, female rats were exposed to a variety of compound stressors-including restraint, shaking, food and water deprivation, crowding, forced swimming, and wet bedding-over a 21-day gestation period. The intervention involved administering ZGP concentrate at different stages: the prevention group received ZGP (1.89 g/kg/day) via gavage throughout the entire pregnancy, while the treatment group was administered the same dosage by gavage starting from the 21st day postpartum for a duration of 21 days. The experimental results demonstrated that the offspring of the model group exhibited significant reductions in epididymal and testicular indices, spermatogenesis function, sperm density, and motility rate. Additionally, testicular tissue displayed signs of oxidative stress, evidenced by decreased SOD activity and increased MDA content, as well as abnormal apoptotic signaling. This was characterized by reduced expression of B-cell lymphoma 2 (Bcl-2) and connexin43 and increased expression of BCL-2 associated X protein (Bax) and Caspase-3. The administration of ZGP was found to effectively reverse these abnormalities and significantly ameliorate sperm damage in the offspring. The underlying mechanism is closely associated with the enhancement of the body’s antioxidant capacity and the inhibition of apoptotic pathways in testicular tissue cells [32]. This study provides a novel experimental foundation and theoretical support for the transgenerational protective effects of TCM compounds in the field of reproductive health.

#### 2.1.4. Yishen Tongluo Formula

The Yishen Tongluo Formula (YSTLF), composed of Cuscutae Semen (*Cuscuta chinensis* Lam.), Rehmanniae Radix Praeparata (*Rehmannia glutinosa* (Gaertn.) DC.), Epimedii Folium (*Epimedium brevicornu* Maxim.), Astragali Radix (*Astragalus membranaceus* (Fisch.) Bge.), Salviae Miltiorrhizae Radix et Rhizoma (*Salvia miltiorrhiza* Bunge), Hirudo (*Hirudo nipponica* Whitman), and Achyranthis Bidentatae Radix (*Achyranthes bidentata* Bl.), is a commonly employed therapeutic prescription for the treatment of liver-kidney Yin deficiency and blood stasis syndrome in diabetic nephropathy. Research has substantiated the formula’s efficacy in regulating blood glucose levels, alleviating oxidative stress, inhibiting inflammation, and facilitating renal function recovery [63]. In recent years, there has been a growing interest in its potential role in enhancing male reproductive function.

Pior studies have demonstrated that the YSTLF significantly reduces the sperm DNA fragmentation index (DFI) [64], repairs sperm DNA damage [65], and inhibits aberrant DNA methylation [66], thereby indicating its potential to preserve sperm genetic integrity. The YSTLF holds promising potential for mitigating reproductive damage induced by environmental pollutants. The reproductive toxicity of microplastics, including polystyrene microplastics, has gained increasing recognition [67,68], however, effective prevention and control strategies remain limited. In a study conducted by Zhang et al., it was found that in a rat model where polystyrene microplastics induced sperm DNA damage, the administration of YSTLF (1.044 g/mL, 1 mL/100 g body weight) via gavage significantly decreased DFI, MDA, and nitric oxide (NO) levels, while enhancing SOD activity and adenosine triphosphate (ATP) content in testicular tissue. These findings suggest that the decoction may exert a protective effect by augmenting the body’s antioxidant capacity [33]. Furthermore, the YSTLF has shown potential in mitigating reproductive damage induced by benzo[a]pyrene and other common environmental pollutants. Zhao et al. developed a model of sperm DNA damage in Wistar rats induced by benzo[a]pyrene. Their findings indicated that following intragastric administration of YSTLF (1.2 g/mL, 1 mL/100 g body weight, for 30 consecutive days), there was a significant reduction in DFI, testicular MDA, and NO levels in the model rats. Concurrently, an increase in SOD activity, ATP, and serum testosterone levels was observed, while levels of follicle-stimulating hormone (FSH) and luteinizing hormone (LH) decreased. These results suggest that the formula may ameliorate pollutant-induced damage to the male reproductive system by reducing oxidative stress and modulating reproductive endocrine function [34].

Clinical research has further corroborated the efficacy of YSTLF in treating male infertility. The study conducted by Li et al. demonstrated that a 12-week treatment with YSTLF resulted in significant improvements in the proportion of forward motile sperm, the rate of sperm with normal morphology, and key biochemical markers, including SOD, acid phosphatase, zinc, fructose, and α-glucosidase in seminal plasma. Additionally, there was a significant reduction in MDA levels, suggesting that this formulation may improve sperm quality by optimizing the seminal plasma microenvironment and enhancing antioxidant capacity [69]. Similarly, research conducted by Lu et al. indicated that the oral administration of Yishen Tongluo granules significantly improved semen volume, sperm concentration, total motility, acrosin activity, normal morphology rate, and the total antioxidant capacity of seminal plasma. Furthermore, it reduced the deformity rate of DFI, levels of ROS and MDA in seminal plasma, and FSH in serum. These findings further suggest that the prescription may contribute to fertility protection by enhancing the antioxidant capacity of testicular spermatogenic cells and mature sperm [70].

#### 2.1.5. Qilin Pill

The Qilin Pill (QLP) is a complex formulation comprising Polygoni Multiflori Radix (*Polygonum multiflorum* Thunb.), Ecliptae Herba (*Eclipta prostrata* L.), Epimedii Folium (*Epimedium brevicornu* Maxim.), Cuscutae Semen (*Cuscuta chinensis* Lam.), Cistanches Herba (*Cistanche deserticola* Y.C.Ma), Codonopsis Radix (*Codonopsis pilosula* (Franch.) Nannf.), Curcumae Radix (*Curcuma longa* L.), Lycii Fructus (*Lycium barbarum* L.), Rubi Fructus (*Rubus chingii* Hu), Dioscoreae Rhizoma (*Dioscorea opposita* Thunb.), Salviae Miltiorrhizae Radix et Rhizoma (*Salvia miltiorrhiza* Bunge), Astragali Radix (*Astragalus membranaceus* (Fisch) Bge.), Paeoniae Radix Alba (*Paeonia lactiflora* Pall.), Citri Reticulatae Pericarpium Viride (*Citrus reticulata* Blanco) and Mori Fructus (*Morus alba* L.).

Its protective effects on male reproductive function have been systematically validated in rat models. In a study conducted by Zhang et al., a rat model of oligoasthenospermia was induced using *Tripterygium wilfordii* polyglycoside (40 mg/kg, administered via continuous gavage for four weeks). The intervention involved administering QLP at dosages of 1.62 g/kg and 3.24 g/kg, also via continuous gavage, over a period of 60 days. The findings indicated that QLP significantly improved sperm concentration and motility in the model rats and mitigated pathological and DNA damage in testicular tissue. Mechanistically, the compound exerts its reproductive protective effects by modulating the expression of key factors such as Bcl-2, Bax, cytochrome c (Cyt c), caspase-9, and caspase-3, thereby inhibiting the mitochondrial-mediated apoptotic pathway in testicular cells. [35]. Subsequent investigations have demonstrated that the administration of QLP can restore serum levels of FSH, LH, prolactin, free testosterone (FT), and sex hormone-binding globulin in rat models. Additionally, it decreases the concentrations of ROS and MDA in testicular tissue, enhances the activity of SOD, and upregulates the expression of the testicular-specific serine kinase 2 (TSSK2) gene. These findings indicate that QLP may holistically enhance sperm quality and testicular tissue architecture by modulating reproductive endocrine function, enhancing antioxidant capacity, and promoting the expression of genes associated with spermatogenesis [36].

Numerous multicenter randomized controlled trials and systematic studies have substantiated its clinical efficacy in treating idiopathic oligoasthenospermia. In a clinical setting, researchers administered QLP at a dosage of 6 g per session, three times daily for a duration of 12 weeks to patients diagnosed with idiopathic oligoasthenospermia. The results revealed significant improvements in semen parameters, including sperm motility, total sperm count, and progressive sperm movement, observed at 4, 8, and 12 weeks following treatment. Notably, the effects became more pronounced with prolonged treatment duration [71,72,73].

### 2.2. Prescriptions for Warm and Invigorate Kidney Yang

The warm and invigorate kidney yang formula is primarily focuses on warming and tonifying kidney Yang, as well as invigorating Yang Qi. This formulation is particularly effective in treating male infertility associated with kidney Yang deficiency. It has been shown to improve microcirculation within the reproductive system, enhance antioxidant capacity, and promote testicular spermatogenesis and sperm motility.

#### 2.2.1. Shenrong Pill

The Shenrong Pill (SRP), a formulation consisting of 18 traditional Chinese medicinal ingredients such as Ginseng Radix Rubra (*Panax ginseng* C.A.Mey.), Cervi Cornu Pantotrichum (*Cervus nippon* Temminck), Morindae Officinalis Radix (*Morinda officinalis* How), Cinnamomi Cortex (*Cinnamomum cassia* Presl), Cistanches Herba (*Cistanche deserticola* Y.C.Ma), Lycii Fructus (*Lycium barbarum* L.), Cuscutae Semen (*Cuscuta chinensis* Lam.), Rehmanniae Radix Praeparata (*Rehmannia glutinosa* (Gaertn.) DC.), Poria (*Poria cocos* (Schw.) Wolf), Astragali Radix (*Astragalus membranaceus* (Fisch.) Bge.), Paeoniae Radix Alba (*Paeonia lactiflora* Pall.), Atractylodis Macrocephalae Rhizoma (*Atractylodes macrocephala* Koidz.), Citri Reticulatae Pericarpium (*Citrus reticulata* Blanco), Angelicae Sinensis Radix (*Angelica sinensis* (Oliv.) Diels), Achyranthis Bidentatae Radix (*Achyranthes bidentata* Bl.), Dioscoreae Rhizoma (*Dioscorea opposita* Thunb.), Foeniculi Fructus (*Foeniculum vulgare* Mill.) and Glycyrrhizae Radix et Rhizoma (*Glycyrrhiza uralensis* Fisch.), is known for its effects in nourishing Yin, tonifying the kidney, benefiting essence, and strengthening Yang.

To investigate the protective effects of SRP against oxidative stress -induced damage in testicular stromal cells, Tang et al. established an oxidative stress model by exposing mouse testicular stromal cells (TM3 cells) to 600 µmol/L H_2_O_2_. The study incorporated interventions using SRP-containing serum at concentrations of 7.5%, 10% and 12.5%. The findings indicated a significant increase in the levels of lipid peroxidation (LPO) products and MDA in TM3 cells after a 24-h exposure to H_2_O_2_, accompanied by a marked decrease in the activities of critical antioxidant enzymes, including superoxide dismutase-1 (SOD-1), catalase (CAT), and glutathione peroxidase (GSH-Px). This suggests that the oxidative stress cell model was successfully established. Subsequent intervention with SRP-containing serum notably mitigated the oxidative stress effects induced by H_2_O_2_, as evidenced by reduced LPO and MDA levels and increased activities of SOD-1, CAT, and GSH-Px. These results imply that SRP-containing serum effectively attenuates oxidative stress injury by enhancing the antioxidant capacity of TM3 cells, thereby providing a protective effect on reproductive health [37].

#### 2.2.2. Jinkui Shenqi Pill

The Jinkui Shenqi Pill (JKSQP), derived from the “Synopsis of the Golden Chamber” comprises eight medicinal components: Rehmanniae Radix Praeparata (*Rehmannia glutinosa* (Gaertn.) DC.), Dioscoreae Rhizoma (*Dioscorea opposita* Thunb.), Cornus Fructus (*Cornus officinalis* Sieb. et Zucc.), Aconiti Lateralis Radix Praeparata (*Aconitum carmichaelii* Debx.), Cinnamomi Ramulus (*Cinnamomum cassia* Presl), Alismatis Rhizoma (*Alisma orientale* (Sam.) Juzep.), Poria (*Poria cocos* (Schw.) Wolf), and Moutan Cortex (*Paeonia suffruticosa* Andr.). The JKSQP, historically recognized as “the ancestor of Tonifying Kidney Yang for thousands of years,” is extensively utilized in the treatment of oligoasthenospermia associated with kidney Yang deficiency.

In a study conducted by Li et al., the therapeutic effects of JKSQP were evaluated using a rat model of kidney yang deficiency induced by cortisone administration. The experimental protocol involved the intragastric administration of JKSQP at a concentration of 0.5 g/mL, based on the crude drug volume, via intragastric administration at a dosage of 10 mL/kg per day over a period of 20 consecutive days. The findings indicated that compared to the control group, the model group exhibited a significant reduction in body weight, serum testosterone levels, and SOD activity, along with an increase in MDA levels. Following treatment with JKSQP, these parameters showed marked improvement, suggesting its potential role in modulating endocrine function and mitigating oxidative stress [38]. Additionally, Jiang et al. investigated the role and underlying mechanisms of JKSQP in a cyclophosphamide-induced oligoasthenospermia model. In this study, the intragastric administration of JKSQP at a dosage of 1.2 g/kg led to a significant increase in the testicular organ index, sperm quality parameters, and serum testosterone levels in mice. Mechanistic investigations revealed that this formulation enhances SOD activity and reduces MDA content by modulating the expression of key genes within the nuclear factor E2-related factor 2 (Nrf2) signaling pathway. This finding elucidates the pharmacological basis for its protective effects on testicular tissue via the antioxidant pathway [39].

In summary, JKSQP not only ameliorates reproductive endocrine dysfunction associated with kidney yang deficiency syndrome but also enhances the body’s antioxidant capacity and mitigates oxidative damage to testicular tissue through activation of the Nrf2 pathway. This provides robust experimental foundation for its potential clinical application in the treatment of male infertility.

### 2.3. Prescriptions for Replenish Qi and Nourish Blood-Alongside

The Yiqi yangxue formula, which primarily functions to tonify Qi and blood and harmonize the viscera, has been shown to enhance the circulation of Qi and blood within the reproductive system and augment the body’s antioxidant capacity. It is particularly beneficial for addressing male infertility associated with deficiencies in both Qi and blood.

#### 2.3.1. Bazhen Decoction

The Bazhen Decoction (BZD) is composed of several principal ingredients: Ginseng Radix et Rhizoma (*Panax ginseng* C. A. Mey.), Rehmanniae Radix Praeparata (*Rehmannia glutinosa* (Gaertn.) DC.), Atractylodis Macrocephalae Rhizoma (*Atractylodes macrocephala* Koidz.), Poria (*Poria cocos* (Schw.) Wolf), Angelicae Sinensis Radix (*Angelica sinensis* (Oliv.) Diels), Paeoniae Radix Alba (*Paeonia lactiflora* Pall.), Chuanxiong Rhizoma (*Ligusticum chuanxiong* Hort.), and Glycyrrhizae Radix et Rhizoma (*Glycyrrhiza uralensis* Fisch.). This traditional formulation is employed to tonify Qi and the spleen, nourish the blood, and activate blood circulation. Of particular interest is ferulic acid, a phenolic acid present in *Angelica sinensis*, which demonstrates significant antioxidant properties by effectively neutralizing free radicals in the reproductive system and maintaining the integrity of sperm membrane structures. Furthermore, the active constituents in Rehmanniae Radix Praeparata and Chuanxiong Rhizoma work synergistically to collaborate Qi and blood, enhance microcirculation within the reproductive system, and mitigate oxidative stress-induced damage.

In prior research, reproductive system indices were compared between 15-month-old and 2-month-old mice. The findings indicated a significant increase in serum MDA levels in the older mice, accompanied by a notable decrease in SOD activity and testosterone content. Subsequent investigations revealed that continuous intragastric administration of BZD, at a dosage of 2.5 g/kg of crude drug for four weeks, resulted in significant improvements in these parameters in the older mice. This suggests that BZD may exert a protective effect on male reproductive function by mitigating oxidative stress damage in testicular tissue, thereby inhibiting excessive apoptosis of spermatogenic cells [40].

#### 2.3.2. Danggui Buxue Decoction

Danggui Buxue Decoction (DBD), which originated from the theory of internal and external injuries proposed by Li Dongyuan during the Jin and Yuan Dynasties, consists of Astragali Radix (*Astragalus membranaceus* (Fisch.) Bge.) and Angelicae Sinensis Radix (*Angelica sinensis* (Oliv.) Diels) in a 5:1 ratio. It is a TCM prescription renowned for its capacity to enhance Qi and promote blood generation.

In order to investigate the protective effects of this prescription on testicular injury induced by a high-fat diet, Qiu et al. established a rat model of testicular injury triggered by a high-fat diet. The rats were administered DBD intragastrically at dosages of 6 g/kg/day and 12 g/kg/day, respectively. The results demonstrated that, relative to the control group, the high-fat diet group exhibited a significant decrease in both total sperm count and sperm motility. Additionally, there was an observed increase in MDA content within the testicular tissue and a decrease in SOD activity. Histological analysis using hematoxylin and eosin staining revealed that a high-fat diet resulted in atrophy and degeneration of the testicular seminiferous tubules, along with a loss of seminiferous epithelium. Moreover, TUNEL assay results indicated a statistically significant increase in the number of apoptotic bodies and the apoptosis rate in the testicular tissue of rats subjected to the high-fat diet. Following DBD administration, there was a marked improvement in sperm motility and total sperm count in both the low-dose and high-dose rat groups, with values approaching normal levels. Simultaneously, a notable decrease in MAD content was observed in the testicular tissue, accompanied by a significant increase in SOD activity. Additionally, the decoction effectively maintained the structural integrity of testicular tissue, inhibited the formation of apoptotic bodies, and reduced the rate of cellular apoptosis. These results suggest that DBD provides a considerable protective effect against testicular injury induced by a high-fat diet in rats. The underlying mechanism is likely related to improvements in testicular morphology and structure, restoration of reproductive function, enhancement of antioxidant capacity, and inhibition of testicular cell apoptosis [41].

In conclusion, the effectiveness of this TCM compound in enhancing male reproductive function is primarily attributed to its natural constituents, which possess well-defined antioxidant properties. To further elucidate its material basis and mechanism of action, a systematic classification and analysis of the key natural active ingredients in related compounds will be conducted.

## 3. Natural Active Ingredients for Improving Male Sterility

**Figure 2:**
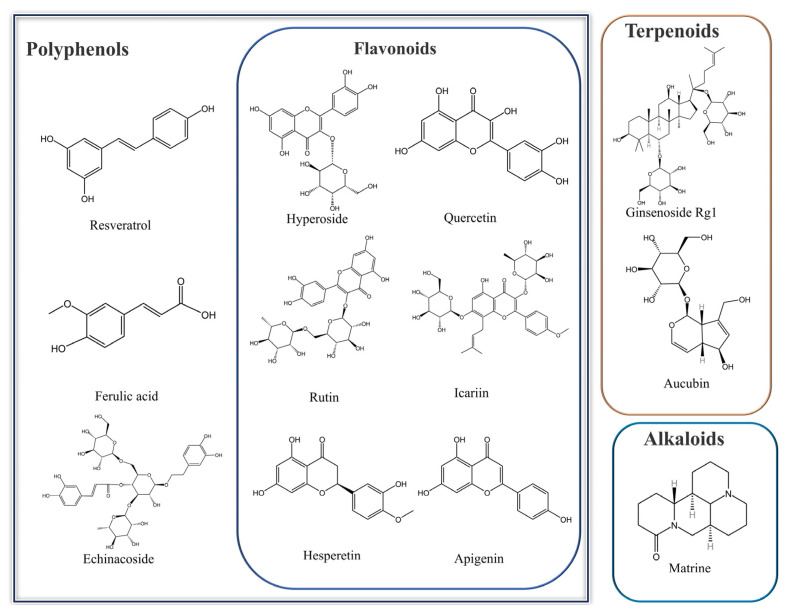
Chemical structures of representative TCM-derived bioactive compounds.

**Figure 3:**
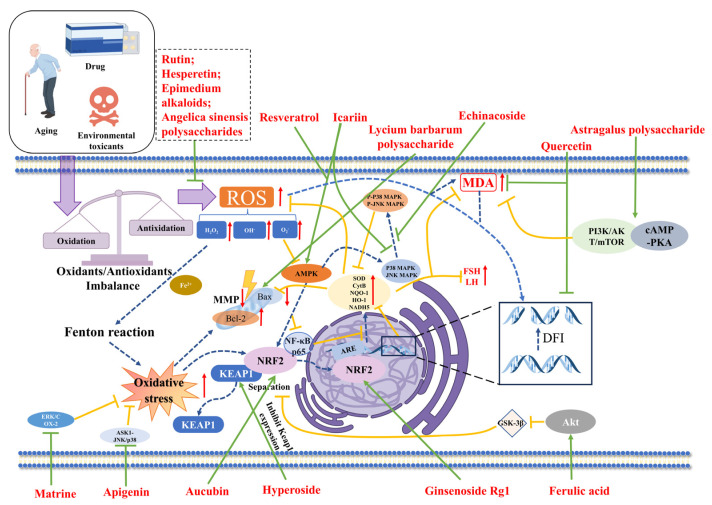
Effective components of TCM improve reproductive related signaling pathways in male mammals. ROS: Reactive oxygen species; NRF2: NF-E2 p45-related factor 2; Keap1: Kelch-like ECH-associated protein 1; SOD: Superoxide dismutase; CAT: Catalase; HO-1: Heme Oxygenase-1; ROS: Reactive oxygen species; NQO-1: NAD (P) H quinone dehydrogenase 1; ARE: AU-rich element; P53: Cellular tumor antigen p53; P38 MAPK: P38 mitogen-activated protein kinase; JNK: c-Jun N-terminal kinase; AMPK: Adenosine 5‘-monophosphate (AMP) -activated protein kinase; Bax: Bcl-2 Assaciated X protein; Bcl-2: B cell lym-phoma 2; DFI: DNA Fragmentation Inde.

Natural active ingredients constitute the “functional unit” of TCM compounds, underpinning their efficacy and forming the core material basis for their clinical pharmacological effects. The diverse array of ingredients within a TCM compound fulfills specific roles, dictated by their unique chemical structures (Figure 2). Extensive research has demonstrated that natural active components derived from various TCM hold significant potential in enhancing the reproductive function of male mammals (Table 2) through the modulation of oxidative stress signaling pathways (Figure 3).

### 3.1. Polyphenols

Polyphenols, characterized by their phenolic hydroxyl groups, exhibit robust antioxidant, anti-inflammatory, and anti-apoptotic properties and flavonoids constitute one of the most important subclasses of polyphenols. In the context of male reproductive health, these compounds mitigate testicular damage and sperm dysfunction caused by chemical toxins, heavy metals, and pollutants by scavenging ROS and modulating oxidative stress pathways.

Polyphenols exhibit distinctive pharmacokinetic properties, characterized by limited absorption efficiency, rapid metabolic transformation, suboptimal tissue targeting, and diverse excretion pathways. Predominantly present as glycosides in nature, polyphenols necessitate hydrolysis into aglycones by intestinal glycosidases for absorption, primarily occurring in the upper small intestine; fractions that remain unabsorbed may undergo further metabolism by gut microbiota for secondary utilization. Nevertheless, their overall oral bioavailability is generally low, attributed to the combined effects of hepatic first-pass metabolism and intestinal efflux transporters. Following absorption, polyphenols predominantly accumulate in metabolically active organs such as the liver, kidneys, and intestines, where they are susceptible to phase II conjugation reactions (e.g., glucuronidation and sulfation) in the liver, resulting in the formation of more water-soluble metabolites. Both the parent polyphenols and their metabolites exhibit relatively short elimination half-lives, primarily excreted via the kidneys in urine, partially eliminated in feces through biliary excretion, with a minor fraction potentially extending their in vivo retention time through enterohepatic circulation [111,112].

#### 3.1.1. Resveratrol

Resveratrol, a natural polyphenol found in QLP, demonstrates anti-inflammatory, cardioprotective, anti-tumor, neuroprotective, and antioxidant activities [113,114,115,116]. It enhances the activities of antioxidant enzyme such as SOD, CAT, and GSH-Px, neutralizes free radicals, and inhibits lipid peroxidation, thereby safeguarding cellular function [117,118].

Resveratrol has been shown to exert protective effects on male fertility, as evidenced by studies investigating its impact on ferrous ascorbate (FeAA) -induced oxidative stress in bovine sperm. The results indicate that treatment with various concentrations of resveratrol (5, 10, 25, and 50 µmol/L) over a six-hour period significantly counteracted the FeAA -induced reduction in sperm motility and antioxidant indices, including SOD, CAT, and GSH. Furthermore, there was a significant decrease in superoxide production and lipid peroxidation levels in sperm, suggesting that resveratrol effectively mitigates exogenous oxidative damage [74]. Resveratrol demonstrated protective effects at multiple levels in enhancing boar sperm function. Research has shown that the incorporation of 50 µmol/L resveratrol significantly improves phenotypic functional parameters of sperm post-cryopreservation and thawing, including forward motility, plasma membrane and acrosome integrity, and mitochondrial activity [75]. Furthermore, under liquid storage conditions at 17 °C, resveratrol effectively preserves sperm motility and ultrastructural stability [76]. At the mechanistic level, resveratrol enhances the intracellular antioxidant defense system by activating the AMP-activated protein kinase (AMPK) signaling pathway. This activation is demonstrated by an increase in GSH levels, elevated activities of key antioxidant enzymes such as GSH-Px, SOD, and CAT, as well as an increase in AMPK phosphorylation levels. Consequently, these changes collectively enhance the capacity of sperm to withstand oxidative stress [75,76]. These findings elucidate the complex mechanisms by which resveratrol safeguards boar sperm function, encompassing both functional maintenance and molecular regulation.

#### 3.1.2. Ferulic Acid

Ferulic acid, a polyphenolic compound commonly found in Chinese herbal medicine, exhibits significant antioxidant activity [119]. It is an ingredient in traditional Chinese herbal formulations such as WZYZP, BZBSP, ZGP, QLP, SRP, BZD, DBD, and YSTLF.

In models of testicular dysfunction induced by diabetes, ferulic acid has demonstrated a notable reparative effect. Streptozotocin-induced diabetic rats display characteristic pathological changes, including a reduction in testicular weight, decreased testosterone synthesis, and impaired spermatogenesis. Following the administration of 50 mg/kg of ferulic acid per day, significant improvements were observed in testicular weight, serum testosterone levels, sperm count, and motility, suggesting that ferulic acid exerts a substantial protective effect against diabetes-related reproductive damage [120]. Moreover, ferulic acid exhibited extensive protective effects in various models of testicular toxicity. In instances of testicular injury induced by exposure to lead acetate or sodium arsenate in rats, ferulic acid effectively enhanced the activity of endogenous antioxidant enzymes, thereby improving sperm function [121,122]. Additionally, in cases of testicular injury caused by γ-ray radiation, ferulic acid significantly mitigated radiation-induced damage to the reproductive system by modulating serum testosterone levels, upregulating the expression of testicular SIRT1 protein, and alleviating oxidative stress [123].

The protective effects of ferulic acid are intricately linked to its modulation of multiple signaling pathways. In the diabetic model, ferulic acid mitigates testicular cell apoptosis and enhances tissue antioxidant capacity. These protective effects are mediated through the modulation of the TGF-β1, IL-1β, and protein kinase B (Akt) signaling pathways, thereby ultimately improving testicular function [120].

In the cadmium-induced testicular toxicity model, the mechanism of action of ferulic acid was further elucidated. Exposure to cadmium chloride (CdCl_2_) resulted in cadmium accumulation within testicular tissue, disrupted serum reproductive hormone levels, inhibited Nrf2 expression, and induced oxidative stress, as indicated by elevated MDA and NO levels. Pretreatment with ferulic acid effectively mitigated cadmium toxicity by activating the Nrf2 signaling pathway and restoring the oxidative/antioxidant balance [119].

In vitro experiments further substantiated the beneficial effects of ferulic acid on human sperm function. Research has shown that various concentrations of ferulic acid (0–1.6 mmol/L) can enhance levels of cyclic adenosine monophosphate (cAMP) and cyclic guanosine monophosphate (cGMP) levels in sperm from both fertile individuals and patients with asthenospermia, improve sperm motility, and reduce damage from membrane lipid peroxidation. These findings suggest that ferulic acid directly enhances sperm quality by modulating the cyclic nucleotide signaling system and antioxidant mechanisms [124].

#### 3.1.3. Echinacoside

Echinacoside, a natural phenylethanoid glycoside, serves as a principal active constituent of *Cistanche deserticola*, which is utilized in both BZBSP and SRP [125,126].

In an in vitro cellular study, TM3 cells were exposed to 400 µmol/L of H_2_O_2_ for 4 h to establish an oxidative stress injury model of male germ cells. Upon successful establishment of the model, cells in the model group demonstrated significant detachment and a reduction in cell number. Subsequently, TM3 cells were treated with echinacoside at concentrations of 50, 100, and 200 µmol/L for 24 h. The result indicated that echinacoside enhanced the proliferation of TM3 cells in a concentration-dependent manner following oxidative stress injury [80]. The mitogen-activated protein kinase (MAPK) signaling pathway is known to regulate various physiological processes in organisms, including cell growth, differentiation, and apoptosis. Research has demonstrated that the levels of key signaling molecules, such as c-Jun N-terminal kinase (JNK) and p38 mitogen-activated protein kinase (p38), are closely associated with oxidative stress [127,128,129,130,131]. Western blot analysis demonstrated an upregulation in the expression levels of phosphorylated p38 (p-p38) and phosphorylated JNK (p-JNK) proteins following H_2_O_2_ treatment, indicating the activation of the intracellular oxidative stress response. Subsequent treatment with echinacoside resulted in a reduction in the expression of p-p38 and p-JNK in TM3 cells compared to the H_2_O_2_ treatment group alone, suggesting that echinacoside can mitigate the activation of the MAPK signaling pathway induced by H_2_O_2_ [80].

In an in vivo study, rats subjected to intraperitoneal injection of lead acetate (25 mg/kg) for seven consecutive days exhibited testicular tissue atrophy, reduced sperm count, increased deformity rate, impaired motility, and symptoms of infertility. However, following intragastric administration of echinacoside (50–100 mg/kg) for 30 days, there was a marked improvement in testicular morphology and partial restoration of sperm function. Serum biochemical analysis revealed significant increases in the levels of GSH, LDH, and SOD in rats treated with echinacoside. Subsequent investigations have demonstrated that lead acetate significantly upregulates the expression of p-p38 and p-JNK proteins in rat testis. Intragastric administration of echinacoside significantly reduced p38 and JNK phosphorylation levels in the testis, suggesting its potential to improve spermatogenesis dysfunction caused by oxidative stress. This is achieved by inhibiting p38 and JNK phosphorylation, modulating the MAPK signaling pathway, and enhancing antioxidant enzyme activity [80].

### 3.2. Flavonoids

**Figure 4:**
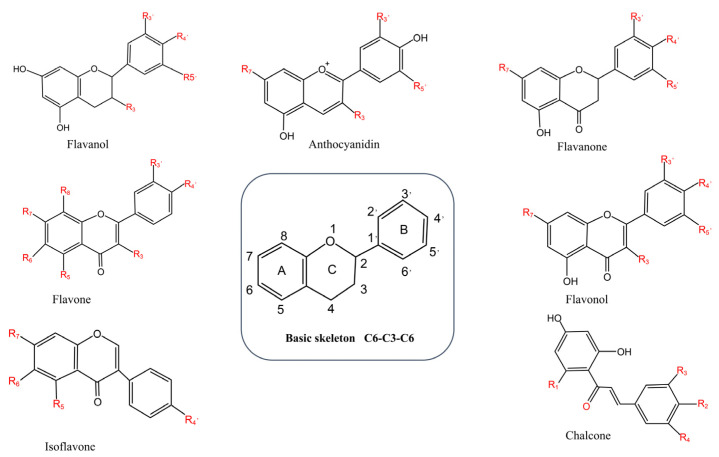
Structure of different groups of flavonoids.

Flavonoids, a class of natural products prevalent in the plant kingdom, are characterized by their polyphenolic structure rich in hydroxyl groups, which exhibit considerable variability and diversity. The biological activity of flavonoids is closely related to their structural features, such as phenolic hydroxyl groups, conjugated systems, and glycosylation (Figure 4). These structural factors critically influence the physicochemical properties of flavonoids, thereby determining their absorption efficacy, bioavailability, distribution, and metabolic transformation behavior in the body; and since the biological activity of flavonoids is closely associated with their absorption efficacy and bioavailability, numerous studies have demonstrated that following absorption in the stomach and small intestine via either active transport or passive diffusion, flavonoids undergo metabolic processes in small intestinal epithelial cells and the liver—such metabolism primarily involves the biotransformation of flavonoids through conjugation with sulfate, glucuronide, or methyl moieties; notably, a subset of flavonoids can also be internalized by colonic microflora, which catabolizes these compounds into phenolic acids and aldehydes, and subsequent to further modification in the liver, these metabolites are transported into the systemic circulation [132]. Flavonoids protect cells by scavenging free radicals, inhibiting ROS production, modulating the endogenous antioxidant enzyme system, and interfering with oxidative stress-related signaling pathways, such as the Nrf2/ARE (AU-rich element) pathway. Furthermore, these compounds can reduce the release of pro-inflammatory mediators, regulate the expression of key proteins involved in the apoptosis pathway, and mitigate cell damage induced by oxidative stress through multiple mechanisms [133].

#### 3.2.1. Hyperoside

Hyperoside, a representative flavonol glycoside also known as quercetin 3-O-beta-D-galactopyranoside, exhibits the classical flavonoid structure of C_6_ (A-ring)-C_3_ (C-ring)-C_6_ (B-ring). Its antioxidant activity is likely due to the presence of hydroxyl groups on the A- and B-rings, in addition to the glycosides attached to the C-ring [134]. This flavanol glycoside compound is derived from dodder (*Cuscuta chinensis* Lam) and other botanical sources and is included in traditional formulations such as WZYZP, BZBSP, ZGP, and QLP. It demonstrates notable anti-inflammatory and antioxidant properties [81,135].

To elucidate the protective effects of hyperoside on the male reproductive system, various research teams have systematically investigated its mechanisms across three levels: animal models, cell experiments, and human sperm studies.

At the cellular level, the study involved exposing mouse spermatocytes (GC-2 cells) to 150 µmol/L of H_2_O_2_ for 2 h to induce an oxidative stress model. The findings revealed a significant decrease in cell viability and the activities of SOD, GSH-Px, and CAT, accompanied by a pronounced increase in MDA content and apoptosis rate. After a 48-h pretreatment with varying concentrations of hyperoside (50, 100, 200 µmol/L), there was a significant improvement in oxidative stress markers. Mechanistic studies demonstrated that hyperoside significantly inhibited the expression of Keap1 (Kelch-like ECH-associated protein 1) protein expression in GC-2 cells, without significantly affecting the protein levels of Nrf2, nuclear Nrf2, and heme oxygenase-1 (HO-1). Hyperoside intervention notably suppressed Keap1 expression, promoted Nrf2 nuclear translocation, and increased HO-1 expression, thereby reducing levels of MDA, ROS, FSH, and LH, effectively mitigating oxidative stress-induced damage in GC-2 cells [82].

In parallel animal studies, oxidative stress injury was induced in the mouse testis through intraperitoneal injection of triptolide (TP). Western blot analysis revealed that, compared to the control group, the expression of Nrf2 in the testes of the model group was significantly reduced. In contrast, treatment with hyperoside significantly enhanced both the expression and nuclear localization of Nrf2. Simultaneously, the expression level of the Keap1 protein was significantly reduced, indicating that hyperoside may alleviate TP-induced testicular tissue damage through activation of the Keap1-Nrf2 signaling pathway [81].

Importantly, hyperoside also exhibits protective effects on human sperm function against oxidative stress in vitro. In a human sperm oxidative stress model induced by 300 µmol/L of H_2_O_2_, total sperm motility was significantly reduced, while the nuclear DFI and LPO levels increased to 1.30 ± 0.19 and 1.71 ± 0.70 times their baseline values, respectively. However, subsequent co-incubation with 100 µmol/L of hyperoside significantly ameliorated the reduction in sperm motility and the elevations in DFI and LPO induced by H_2_O_2_, restoring these parameters to near-normal levels [83]. These results extend the reproductive protective effects of hyperoside from experimental models to human germ cells, providing a strong basis for clinical application.

#### 3.2.2. Quercetin

Quercetin, a bioactive natural compound with the canonical flavonoid structure of C_6_ (A-ring)-C_3_ (C-ring)-C_6_ (B-ring), is a polyhydroxylated flavonoid distinguished by multiple phenolic hydroxyl groups in its chemical structure. These groups impart significant antioxidant properties, enabling it to mitigate cellular damage caused by oxidative stress [136,137,138,139,140,141,142]. As a naturally occurring antioxidant, quercetin is abundant in various medicinal plants, including Ginkgo biloba, Hypericum perforatum, and Canadian elderberry [143]. Within the framework of TCM, quercetin is a prevalent active constituent in formulations such as WZYZP, BZBSP, ZGP, YSTLF, QLP, JKSQP, SRP, BZD, and DBD.

Preliminary in vivo studies have indicated that quercetin may exert a protective effect on reproductive health. In a rat model of CdCl_2_-induced testicular toxicity, quercetin demonstrated significant antioxidant and protective effects. Cadmium exposure led to marked accumulation in serum, testis, and epididymis, accompanied by reduced body and reproductive organ weights, decreased sperm count and motility, and disrupted serum hormone profiles—including declines in gonadotropin-releasing hormone (GnRH), FSH, LH, and testosterone. Furthermore, cadmium increased testicular glucose and lactate levels as well as lactate dehydrogenase activity, indicating altered energy metabolism. Oxidative stress was evident from diminished activities of SOD, CAT, and GSH-Px, along with elevated MDA and H_2_O_2_ levels in testicular tissue [84].

Quercetin co-administration notably attenuated these cadmium-induced alterations. It improved testicular and epididymal weights, restored sperm parameters toward control levels, and partially normalized hormonal levels of GnRH, FSH, LH, and testosterone. Additionally, quercetin modulated testicular energy metabolism by lowering glucose and lactate concentrations and reducing lactate dehydrogenase activity. Importantly, quercetin enhanced the antioxidant defense system, as shown by increased SOD, CAT, and GSH-Px activities and decreased MDA and H_2_O_2_ levels. In the recovery group (observed after cessation of cadmium exposure without quercetin intervention), partial spontaneous improvements in some parameters were noted, but these were less pronounced than the consistent and significant restoration achieved with concurrent quercetin treatment. These findings indicate that quercetin effectively mitigates cadmium-induced testicular damage primarily through antioxidant activity and restoration of metabolic and endocrine function [84].

Notably, the protective effects of quercetin on human sperm have been extensively, highlighting its potential for clinical application. A series of in vitro studies have demonstrated that quercetin effectively enhances human sperm resistance to oxidative stress. Firstly, under physiological conditions, quercetin significantly improves sperm motility in a concentration- and time-dependent manner [85]. Secondly, in a pathological model of leukocytospermia, utilizing sperm samples from patients diagnosed with asthenozoospermia, treatment with 10 µmol/L quercetin for 2 h in vitro increased total sperm motility from 22.54% to 40.54%. This treatment also elevated the levels of cytochrome b (Cyt b) and NADH5 in semen, while reducing H_2_O_2_ levels from 113.02 mmol/L to 72.93 mmol/L. These findings suggest quercetin exerts a dual protective effect by directly scavenging reactive oxygen species and enhancing sperm energy metabolism [86]; Thirdly, in the context of sperm cryopreservation within assisted reproductive technology, the addition of 50 µmol/L quercetin, followed by resuscitation after 7 days of cryopreservation, resulted in significantly higher total sperm motility in the treatment group (33.75 ± 2.93%) compared to the control group (16.5 ± 3.27%). Additionally, there was a notable reduction in MDA levels (1.53 ± 0.6 µmol/L vs. 2.7 ± 0.93 µmol/L), alongside significant enhancements in TAC and the activities of SOD, GSH, CAT. The DFI was significantly decreased, suggesting that quercetin effectively preserves the structural and functional integrity of sperm during freezing [87].

It is noteworthy that the pharmacology of quercetin is complex, and its effects are not universally beneficial. Studies in mammalian models have indicated that high-dose quercetin supplementation alone can induce testicular oxidative stress and adversely affect sperm parameters, underscoring a narrow therapeutic window [144]. Furthermore, quercetin can exhibit pro-oxidant activity under certain conditions, potentially contributing to cellular damage rather than protection [145]. These findings highlight that the supplemental use of quercetin, while promising, requires careful dose consideration to avoid unintended adverse outcomes on reproductive and other physiological systems.

#### 3.2.3. Rutin

Rutin, a bioactive natural flavonoid glycoside derived from quercetin, possesses the canonical C_6_ (A-ring)-C_3_ (C-ring)-C_6_ (B-ring) flavonoid structure and is characterized as a polyhydroxylated flavonoid with multiple phenolic hydroxyl groups on its A- and B-rings. This compound is prevalent in Fagopyrum esculentum Moench, as well as in tea, fruits, and various other plant species [146,147,148,149]. Studies have demonstrated that rutin exhibits a wide array of pharmacological activities, including antioxidant, anticancer, cytoprotective, antiplatelet aggregation, antithrombotic, vascular protective, and anti-inflammatory effects [150]. Importantly, rutin is an active component in several TCM formulations, such as WZYZP, BZBSP, ZGP, YSTLF, QLP, SRP, and BZD.

Sperm cryopreservation is a crucial aspect of sperm banking and assisted reproductive technologies; however, the formation of ice crystals and oxidative stress during the freezing and thawing processes can result in significant damage to sperm structure and function [151,152]. Currently, glycerol egg yolk citrate is the most commonly used cryoprotectant, but its protective efficacy for sperm remains limited [152]. In recent years, researchers have investigated the integration of natural or synthetic active compounds into cryoprotectants to augment their protective efficacy [153,154,155]. In vitro studies using animal (boar) sperm have demonstrated that the incorporation of rutin, at concentrations ranging from 0.2 to 2.0 mmol/L, into cryoprotective solutions can effectively mitigate the accumulation of ROS and the production of MDA in porcine sperm. This is accomplished by enhancing the activities of SOD, CAT, and GSH-Px, thereby significantly improving motility, mitochondrial function, and the integrity of the plasma membrane and acrosome in cryopreserved sperm [77]. Further research has substantiated that supplementing with 0.75–1.0 mmol/L of rutin not only enhances the total and progressive motility of ram epididymal sperm but also effectively inhibits apoptosis. Mechanistic studies have elucidated that rutin treatment increases the TAC and GSH-Px activity of sperm, while also elevating ATP levels, thus improving mitochondrial function and enhancing cell membrane stability [78].

#### 3.2.4. Icariin

Icariin, a bioactive flavonol glycoside derived from *Epimedium* species, possesses the characteristic C_6_ (A-ring)-C_3_ (C-ring)-C_6_ (B-ring) flavonoid structure and is classified as a polyhydroxylated flavonoid with multiple phenolic hydroxyl groups on its A- and B-rings. Owing to its diverse pharmacological properties, this compound has attracted substantial interest across various research fields, particularly in cardiovascular protection, neural regulation, bone metabolism, and the enhancement of reproductive system function [156,157,158,159,160,161]. The pharmacological mechanisms of icariin are complex, involving antioxidant, anti-inflammatory, and immune regulatory activities, which collectively provide a theoretical foundation for its application in male reproductive health [160,162,163,164,165,166]. Notably, icariin is a prominent active ingredient in numerous TCM formulations, such as the BZBSP, YSTLF, and QLP, underscoring its critical role in the treatment of male infertility within TCM.

Within the reproductive system, the mechanism of action of icariin is intricately associated with AMPK, a central regulator of energy metabolism. AMPK served as a sensor of cellular energy status and plays a direct role in regulating spermatogenesis within testicular tissue. Studies have shown that AMPK activation leads to a decrease in ROS production and LPO, thereby improving sperm motility [167,168,169,170]. In contrast, mice lacking the *AMPK* α1 gene exhibit impaired spermatogenesis and a reduced number of mature sperm in the epididymis, highlighting the essential role of AMPK in maintaining normal reproductive function [171]. Furthermore, research has demonstrated that icariin can inhibit the nuclear translocation of nuclear factor kappa B p65 (NF-κB p65) by activating the AMPK signaling pathway, thereby reducing the inflammatory response in testicular tissue. Simultaneously, icariin enhances the antioxidant response mediated by Nrf2, collectively improving testicular function. Additional mechanistic studies have indicated that the protective effect of icariin on testicular function in type 1 diabetic mice is significantly diminished when AMPK is antagonized by dorsomorphin or when Nrf2 expression is inhibited through gene knockdown technology. The evidence substantiates that the AMPK-Nrf2 signaling pathway serves as the principal mechanism by which icariin preserves the reproductive function in male mammals [88].

Additionally, icariin has exhibited protective capabilities against male reproductive damage induced by environmental pollutants. Di (2-ethylhexyl) phthalate (DEHP), a common endocrine disruptor, has been documented to trigger apoptosis in Leydig cells, suppress the expression of critical enzymes involved in steroidogenesis, and subsequently reduce testosterone levels and epididymal sperm count, thereby impairing male reproductive function [89]. Experimental findings revealed that exposure to 1 mM DEHP for 12 h significantly elevated ROS levels in mouse Leydig cells, disrupted mitochondrial membrane potential (MMP), and promoted cellular apoptosis. Conversely, pretreatment with 1 µg/mL icariin for 3 h significantly diminished ROS production, maintained mitochondrial functional integrity, and effectively alleviated testicular damage induced by DEHP [54].

Notably, the protective effect of icariin has been substantiated in studies involving human sperm. In a model of oxidative stress in human sperm induced by Fenton reagent (FeSO_4_/H_2_O_2_), the DFI of sperm exposed to 10–60% Fenton reagent for 45 min was significantly elevated compared to the control group. Further investigations have shown that treatment with a 30% Fenton reagent leads to a decrease in the activities of LDH and SOD in sperm, while the level of MDA, a byproduct of lipid peroxidation, also diminishes. Preincubation with 0.001–0.100 µg/mL of icariin in human sperm was observed to restore the activities of LDH and SOD and further reduce MDA levels [172].

#### 3.2.5. Hesperetin

Hesperetin, a primary bioactive dihydroflavonoid aglycone derived from citrus fruits, is a natural flavanone compound and the aglycone of hesperidin. It possesses the canonical C_6_ (A-ring)-C_3_ (C-ring)-C_6_ (B-ring) flavonoid backbone and is structurally characterized as a polyhydroxylated dihydroflavonoid with phenolic hydroxyl groups at the 5 and 7 positions of the A-ring and the 4′ position of the B-ring. This compound is abundant in citrus fruits and is also a component of the traditional Chinese medicinal formula BZD. Extensive research has elucidated the diverse biological activities of hesperetin, encompassing antioxidant, anti-inflammatory, and antitumor properties [173,174,175,176,177,178,179,180,181]. Notably, in a streptozotocin (STZ) -induced diabetic rat model, hesperetin has demonstrated significant antihyperglycemic, antihyperlipidemic, and antioxidant effects [182]. It has been shown to inhibit oxidative stress, neuroinflammation, and apoptosis in the retinas of diabetic rats [183].

Concerning the male reproductive system, hesperetin exhibits potential protective effects. In vivo studies have substantiated its significant ameliorative impact on testicular injury in diabetic Wistar rats. In these studies, diabetic model rats were administered an oral dose of 50 mg/kg hesperetin daily for 46 days. The results indicated that hesperetin not only improved systemic metabolic indices and increased serum testosterone levels in the rats but also significantly reduced oxidative stress markers, such as MDA and ROS. This reduction was achieved by enhancing the antioxidant defense mechanisms within the testicular tissue, including MMP, GSH, CAT, and GSH-Px activities. Furthermore, hesperetin demonstrated significant anti-inflammatory and anti-apoptotic properties, characterized by the downregulation of inflammatory factor expression, a decrease in the DFI, and reduced caspase-3 activity, thus significantly enhancing spermatogenesis [90].

In vitro studies have further corroborated the potential utility of hesperidin in the cryopreservation of human sperm. The results suggest that the inclusion of 20 µmol/L hesperetin in the cryoprotective medium substantially improves the survival rate, vitality, and normal morphology of thawed sperm. Mechanistic studies reveal that hesperetin significantly diminishes ROS and LPO levels in thawed human sperm, while increasing the proportion of sperm with intact plasma membranes, thereby effectively maintaining the structural and functional integrity of the sperm [79].

#### 3.2.6. Apigenin

Apigenin, a bioactive flavone aglycone derived from edible plants such as celery, onion, and chamomile, possesses the canonical C_6_ (A-ring)-C_3_ (C-ring)-C_6_ (B-ring) flavonoid structure, characterized by 5,7-dihydroxyl groups on the A-ring and a 4′-hydroxyl group on the B-ring. This distinctive structural configuration confers apigenin with favorable lipophilicity and cell membrane permeability. Recent studies have demonstrated that apigenin exhibits a diverse array of biological activities, including antioxidant, anti-inflammatory, antiviral, and anticancer properties [184]. Furthermore, apigenin is a constituent of various TCM formulations, such as WAYZP, BZBSP, ZGP, QLP, and DBD.

In vivo animal research conducted by Dang et al. explored he protective effects of apigenin administered via gavage at doses of 234 mg/kg and 468 mg/kg on sperm quality deterioration and testicular damage in Sprague Dawley rats exposed to subchronic acrylonitrile. The results indicated that apigenin significantly improved sperm concentration, motility, and MMP, while reducing ROS and MDA levels in sperm. Additionally, apigenin improved sperm ultrastructure and ameliorated pathological alterations in testicular tissue, while also inhibiting apoptosis in spermatogenic cells. Furthermore, apigenin was found to increase GSH-Px activity and decrease MDA content in testicular tissue [91]. In a separate study, Shi et al. demonstrated that various concentrations of apigenin (117 mg/kg, 234 mg/kg, 351 mg/kg) effectively decreased the rate of sperm deformity and DFI, mitigated damage to testicular tissue, and alleviated oxidative stress in within a rat model of sperm and testicular injury induced by acetonitrile. The underlying mechanism was closely associated with the down-regulation of the ASK1-JNK/p38 signaling pathway and the inhibition of mitochondrial-mediated apoptosis in testicular cells [92].

Additional research has corroborated that apigenin can alleviate oxidative stress-induced damage in cells during sperm cryopreservation. The results demonstrated that the incorporation of 0.2 mmol/L and 0.4 mmol/L apigenin significantly improved the integrity, viability, and motility parameters of bull sperm plasma membranes post-freezing and thawing, while also enhancing the activities of GSH, GSH-Px, CAT, and TAC. Furthermore, apigenin treatment was shown to reduce MDA production and the extent of DNA damage during cryopreservation [185]. Additional studies have indicated that the combination of apigenin with ferulic acid, another potent antioxidant, can synergistically enhance the protective effects against oxidative damage in sperm [93].

### 3.3. Terpenoids

The fundamental structure of terpenoids is characterized by the presence of five-carbon isoprene units (C_5_H_8_). Based on the number of isoprene units in their molecular structure, terpenoids are classified into monoterpenes, sesquiterpenes, diterpenoids, ester terpenes, triterpenes, tetraterpenes, and polyterpenes [185,186]. Terpenoids from different subclasses exhibit diverse biological activities due to their structural variations [187], demonstrating significant potential in antioxidant, anti-inflammatory, and cytoprotective activities, thereby constituting a crucial component of pharmacological research in TCM [188,189].

#### 3.3.1. Ginsenoside Rg1

Ginsenoside Rg1, a principal anti-aging component of *Panax ginseng*, is present in formulations such as BZBSP, SRP, and BZD. This compound plays a crucial role in mitigating stem cell senescence and safeguarding immune organs, the hematopoietic system, and the central nervous system through its antioxidant and anti-inflammatory properties. Moreover, it exhibits a significant antagonistic effect on aging processes induced by D-gal [190].

In a D-gal-induced mouse model of testicular aging, Ginsenoside Rg1 intervention significantly ameliorated oxidative stress markers, including increased SOD activity and TAC, alongside decreased MDA levels, indicating protection against testicular dysfunction [94,95]. Mechanistically, Ginsenoside Rg1 was shown to activate the Nrf2/HO-1 signaling pathway, reversing D-gal-induced suppression of Nrf2 and enhancing the expression of downstream antioxidant proteins such as HO-1, Quinone oxidoreductase 1 (NQO1), GCLC, and GCLM. This demonstrates that Ginsenoside Rg1 alleviates oxidative stress and preserves testicular function primarily through upregulation of the Nrf2-mediated antioxidant defense system [96].

#### 3.3.2. Aucubin

Aucubin, an iridoid compound derived from *Rehmannia glutinosa*, is a common constituent in various traditional Chinese medicinal formulations, such as WZYZP, BZBSP, ZGP, YSTLF, SRP, JKSQP, and BZD. Contemporary research has substantiated the potent antioxidant properties of aucubin [191].

In previous studies, the mouse testicular Sertoli cell line TM4 was utilized as an in vitro model. Sertoli cells are integral to the blood-testis barrier, providing a crucial microenvironment for germ cell development. As a result, the TM4 cell line is widely used in research focused on testicular injury, blood-testis barrier integrity, and related signaling pathways [192,193,194]. The in vitro experimental findings revealed that treatment with TP markedly reduced nuclear Nrf2 protein levels in TM4 cells. However, intervention with aucubin enhanced the stability of Nrf2 protein, promoted its nuclear translocation, and subsequently increased the expression of downstream antioxidant proteins NQO1 and HO-1, thereby alleviating oxidative stress-induced damage. It is noteworthy that the silencing of the gene via transient transfection with Nrf2 siRNA, significantly attenuated the protective effects of aucubin against TP-induced apoptosis, as well as its activation effects on NQO1 and HO-1. This observation further substantiates that the antioxidant effects of aucubin are dependent on the activation of the Nrf2 signaling pathway [97].

In a mouse model of TP-induced testicular injury (TP dose: 120 µg/kg, administered via intraperitoneal injection for two weeks), researchers evaluated the protective efficacy of aucubin. The experimental group animals received pretreatment with varying doses of aucubin (5, 10, and 20 mg/kg) one hour prior to TP administration. The results demonstrated that mice in the model group exhibited testicular atrophy, disruption of the blood-testis barrier, increased levels of ROS, and spermatogenic dysfunction, accompanied by a reduction in sperm count and vitality [97]. Further investigations revealed that in the TP-induced oligoasthenospermia model, pretreatment with aucubin inhibited the increase in ROS and MDA content in testicular tissue in a concentration-dependent manner, and reverse the decline in glutathione and total glutathione activities. TUNEL analysis demonstrated that aucubin significantly reduced the number of TUNEL-positive testicular cells, indicating attenuation of TP-induced DNA fragmentation and apoptosis. Mechanistic investigations revealed that TP compromised the antioxidant defense system by upregulating Keap1 and inhibiting Nrf2 expression. Conversely, aucubin exerted a protective effect by activating the Nrf2 signaling pathway and counteracting the ROS/JNK-dependent mitochondrial apoptosis pathway induced by TP [98].

### 3.4. Alkaloids

Alkaloids, a class of kingdomnitrogen-containing basic organic compounds, are widely distributed across the plant kingdom. They can be classified based on their chemical structures into categories such as organic amines, terpenes, pyridines, isoquinolines, indoles, pyrrolidines, steroids, imidazoles, and purines.

#### 3.4.1. Epimedium Alkaloids

Epimedium alkaloids, a group of secondary metabolites with diverse structures and distinct physiological activities, are predominantly found in *Epimedium* species. These alkaloids, primarily centered around isoquinoline compounds, are essential constituents of TCM formulations, including BZBSP, YSTLF, and QLP.

In normal male mice, Epimedium alkaloids exhibited no significant reproductive toxicity across all tested doses. Conversely, in mice with cyclophosphamide-induced reproductive damage, Epimedium alkaloid intervention significantly increased epididymal and testicular coefficients, improved sperm count and viability, reduced sperm malformation, enhanced testicular SOD activity, decreased MDA levels, elevated serum testosterone, and increased the Bcl-2/Bax ratio, indicating anti-apoptotic effects [99].

#### 3.4.2. Matrine

Matrine, a quinoline alkaloid, is predominantly extracted and isolated from the dried roots, fruits, and other components of *Sophora japonica* L. through the use of organic solvents [195]. This compound is a common constituent in various TCM formulations, including WZYZP, BZBSP, ZGP, YSTLF, QLP, and SRP. Matrine exhibits a wide range of pharmacological activities, including anti-tumor, anti-inflammatory, hepatoprotective, antiviral, antiarrhythmic, and antibacterial properties [196,197,198,199,200]. Recent studies have demonstrated its significant potential in safeguarding male reproductive function.

Numerous studies have identified oxidative stress as the central mechanism underlying permethrin-induced testicular cytotoxicity. Due to its high lipophilicity, permethrin easily penetrates cell membranes and accumulates in testicular tissue, thereby inducing oxidative stress. This process leads to damage to lipids, proteins, and DNA, ultimately resulting in spermatogenic cell death and sperm abnormalities [201,202]. In a study utilizing a testicular toxicity model in male Wistar albino rats induced by permethrin, matrine exhibited significant protective effects on reproductive health. The research indicated that an intraperitoneal administration of matrine at a dosage of 100 mg/kg significantly alleviated testicular pathological damage, increased sperm count, and decreased sperm deformity rates. At the molecular level, matrine confers its protective effects through multiple pathways: firstly, it effectively upregulates the gene expression of steroidogenic acute regulatory protein (StAR), thereby facilitating testosterone synthesis; secondly, it downregulates the expression of phosphorylated extracellular signal-regulated kinase 1/2 (p-ERK1/2) and cyclooxygenase 2 (COX-2); and thirdly, it augments the cellular antioxidant capacity, providing comprehensive protection against damage induced by exogenous substances in testicular tissue [100].

### 3.5. Polysaccharides

Polysaccharides, which are high molecular weight polymers consisting of more than ten monosaccharide units linked via glycosidic bonds, are widely distributed in plants, animals, microorganisms, and TCM. As a major component of the active ingredients in TCM, polysaccharides have attracted significant attention due to their diverse biological activities, including immune modulation, antioxidant properties, anti-inflammatory effects, and the regulation of intestinal homeostasis [203]. This paper reviews the effects and mechanisms of *Lycium barbarum* polysaccharides, *Angelica sinensis* polysaccharides, and *Astragalus* polysaccharides in enhancing male reproductive function.

#### 3.5.1. *Lycium barbarum* Polysaccharide

*Lycium barbarum* polysaccharide is a polysaccharide compound extracted and purified from the mature fruit of *Lycium barbarum*. It is commonly incorporated in traditional formulations such as WZYZP, BZBSP, ZGP, QLP, and SRP.

In a mouse model of oligoasthenospermia induced by oxidative stress due to cadmium exposure, *Lycium barbarum* polysaccharide exhibited a protective effect on reproductive function. Mice subjected to intragastric administration of cadmium at a dose of 5.0 mg/kg for 35 days, showed a significant reduction in the weight of the testis and epididymis, as well as in sperm concentration. Additionally, there was an increase in the rate of abnormal sperm morphology and apoptosis, an elevated level of MDA in the testis, and a decrease in the activities of SOD and GSH-Px. Following intragastric administration of *Lycium barbarum* polysaccharide at dosages of 10, 33.3, and 100 mg/kg for 7 days, alongside continuous cadmium exposure over 35 days, resulted in a significant amelioration of testicular tissue damage in the mice. This treatment also led to notable improvements in serum testosterone levels and sperm motility, indicating that *Lycium barbarum* polysaccharide may mitigate cadmium-induced reproductive toxicity by enhancing antioxidant capacity [101].

Furthermore, *Lycium barbarum* polysaccharide have been shown to ameliorate reproductive toxicity induced by cyclophosphamide in mice. Cyclophosphamide exposure resulted in decreased SOD activity and increased NO levels in the testes, indicative of oxidative stress. However, intragastric administration of *Lycium barbarum* polysaccharide at doses of 0.2, 0.4, and 0.6 g/kg resulted in a significant increase SOD activity and a decrease in NO levels, thereby underscoring its antioxidant properties [102]. Additionally, studies have shown that a dosage of 10 mg/kg *Lycium barbarum* polysaccharide effectively inhibits the mitochondrial apoptotic pathway in sperm. This suppression of apoptosis, characterized by the stabilization of MMP and a favorable shift in the Bax/Bcl-2 ratio towards cell survival, contributes to the enhanced functional preservation of sperm under conditions of oxidative stress [204,205].

A clinical study demonstrated that the addition of 1000 µg/mL *Lycium barbarum* polysaccharide to the cryopreservation solution significantly ameliorated the oxidative stress status of human sperm post-freezing and thawing. The results showed that the sperm MDA content (18.0 ± 3.6 nmol/mL) and the rate of ROS positive cells (38.1 ± 6.7%) in the *Lycium barbarum* polysaccharide treatment group were markedly lower compared to the glycerin egg yolk citrate group (58.3 ± 10.8%). This indicates that *Lycium barbarum* polysaccharide can effectively mitigate oxidative stress-induced damage to sperm DNA and mitochondrial membranes during the cryopreservation recovery process [103].

#### 3.5.2. *Angelica sinensis* Polysaccharide

*Angelica sinensis* polysaccharides are recognized as significant anti-aging components within *Angelica sinensis* exhibiting the capacity to delay stem cell aging and alleviate the adverse effects of aging agents on various organs [206,207,208,209]. *Angelica sinensis* polysaccharides is frequently incorporated into formulations such as SRP, BZD, and DBD.

In a study conducted by Qiu et al., a mouse model of testicular aging was developed through subcutaneous administration of D-gal (120 mg/kg) over a continuous 42-day period. The intervention group, which received *Angelica sinensis* polysaccharides, was administered intraperitoneal injections of *Angelica sinensis* polysaccharides (140 mg/kg) commencing on the 16th day of the modeling phase and continuing for 27 days. The results indicated that, relative to the aging model group, the *Angelica sinensis* polysaccharides intervention led to a reduction in testicular tissue damage, a less pronounced decline in spermatogenic and stromal cells, and mitigation of decreased serum testosterone levels. Additionally, there was a reduction in the density of SA-β-gal positive cells, an increase in SOD activity and TAC, and a decrease in MDA content. Furthermore, the expression levels of p53 and p21 proteins were significantly downregulated. These findings suggest that *Angelica sinensis* polysaccharides may confer a protective effect on testicular tissue by enhancing antioxidant capacity and inhibiting the expression of proteins associated with aging [104].

In the realm of sperm cryopreservation, empirical studies have revealed that the integration of 2000 mg/L of *Angelica sinensis* polysaccharides into ovine sheep semen diluents significantly enhances sperm motility and acrosome integrity, while concurrently augmenting SOD and TAC activities, and reducing MDA levels. This phenomenon is presumably linked to a diminution in oxidative stress within the spermatozoa [105]. Subsequent in vitro analyses have substantiated that the incorporation of 600 µg/mL of *Angelica sinensis* polysaccharides markedly elevates SOD, CAT activity, and TAC levels in goat sperm following cryopreservation and thawing, while concurrently decreasing ROS and MDA content. These findings underscore the prospective utility of *Angelica sinensis* polysaccharides in optimizing cryopreservation outcomes for spermatozoa [106].

#### 3.5.3. Astragalus Polysaccharides

*Astragalus* polysaccharide, an active constituent derived from *Astragalus membranaceus*, is known for antioxidant, anti-inflammatory, and immunomodulatory properties [210]. It is incorporated in formulations such as YSTLF and DBD.

Xiao et al. employed a variety of technical methodologies to demonstrate that *Astragalus* polysaccharide, when administered at a dosage of 100 mg/kg via gavage, can significantly ameliorate testicular damage in mice induced by cantharidin (1.0 mg/kg). The specific mechanisms involve the activation of the Nrf2-Keap1 antioxidant pathway, characterized by the upregulation of Nrf2/HO-1 and the downregulation of Keap1, to mitigate excessive ROS. Additionally, the expression of autophagy-related proteins, such as Beclin-1 and LC3-II, is inhibited, while the mTOR pathway is activated to reduce excessive autophagy. Concurrently, proteins such as ZO-1 and occludin are upregulated to restore the integrity of the blood-testis barrier, ultimately leading to the restoration of testicular tissue morphology and spermatogenesis [107]. Furthermore, considering the reproductive toxicity induced by dibutyl phthalate (DBP), *Astragalus* polysaccharide, administered at a dosage of 200 mg/kg/day via gavage for eight weeks, exhibit a protective effect by enhancing antioxidant defense mechanisms (evidenced by the upregulation of Nrf2 and SOD expression and reduction of MDA levels), inhibiting cellular apoptosis (through the downregulation of caspase-3 and caspase-9 expression), and activating the PI3K/Akt/mTOR pathway [108].

In vitro studies have shown that *Astragalus* polysaccharide, at concentrations between 0.25 and 1 mg/mL, significantly enhances the total motility, forward motility, acrosome integrity, and mitochondrial membrane potential of boar sperm stored at 4 °C, with the optimal concentration identified as 0.5 mg/mL. The mechanisms responsible for these effects are likely related to the reduction of excessive ROS, the maintenance of protein kinase A (PKA) substrate phosphorylation (p-PKAs) and protein tyrosine phosphorylation levels, and the activation of the cAMP-PKA signaling pathway [109]. Furthermore, another study demonstrated that *Astragalus* polysaccharides at a concentration of 0.5 mg/mL significantly enhance the total motility, motility parameters, acrosome integrity, plasma membrane integrity, and mitochondrial activity of frozen-thawed bovine sperm. This improvement is accompanied by increased activities of CAT, SOD, GSH-Px, and other antioxidant enzymes, along with a reduction in ROS and MDA levels [110].

## 4. Perspective and Conclusions

### 4.1. Perspective

Despite significant advancements in research concerning the enhancement of male reproductive function through TCM and natural active compounds, which provide a substantial theoretical foundation and potential candidates for clinical intervention in idiopathic male infertility, numerous scientific challenges persist in this field. Future research could systematically address these challenges in the following directions:

#### 4.1.1. Systematic Analysis of Synergistic Mechanisms in TCM Compounds

Current studies on TCM compounds, such as the WZYZP and BZBSP, primarily focus on evaluating overall efficacy. However, the synergistic mechanisms among the various herbs and components within these compounds have not been thoroughly elucidated. For example, the potential “dose-time effect” synergistic relationship between *lycium barbarum* polysaccharide and *cuscuta chinensis* lam flavonoids in the WZYZP, particularly in the context of regulating spermatogenesis, remains to be clarified. Future investigations should integrate network pharmacology and spatial metabolomics technologies to construct a multi-dimensional network encompassing “components-targets-pathways-phenotypes,” thereby facilitating a comprehensive analysis of the interactions among key active ingredients within the compound. Simultaneously, the creation of a knockout animal model targeting key genes through CRISPR-Cas9 gene editing technology is crucial for validating and verifying the synergistic effects of core component combinations. This strategy seeks to address the limitations inherent in single-component studies, which often fail to accurately represent the compound’s overall efficacy.

#### 4.1.2. Overcoming the Challenges in Clinical Application of Natural Active Ingredients

Although these ingredients have shown promising protective effects on male reproductive health in vitro and in vivo animal models, their clinical application is impeded by several challenges, the most significant being low bioavailability and lack of specificity. For example, despite apigenin’s favorable lipid solubility, its stability in the gastrointestinal environment is insufficient, resulting in an oral bioavailability of less than 10%. To overcome this limitation, future research could focus on two primary strategies: 1. “Dosage Form Innovation”: The development of novel drug delivery systems, such as nanoemulsions, liposomes, and polymer micelles, has the potential to significantly enhance the solubility, stability, and targeting of natural active compounds. For example, encapsulating icariin within liposomes engineered to target testicular stromal cells could substantially increase its concentration within testicular tissue while minimizing its effects on non-target organs, thereby improving the precision and safety of therapeutic interventions. 2. “Structural Modification”: Another crucial strategy involves the optimization of the physicochemical properties of natural active compounds through rational chemical structure modification. This approach seeks to improve the pharmacological attributes of these compounds, thereby improving their efficacy as therapeutic agents. For instance, the methylation of phenolic hydroxyl groups in quercetin molecules can significantly enhance their chemical stability and membrane permeability, while maintaining their intrinsic antioxidant activity. Such modifications consequently improve the pharmacokinetic properties of quercetin in vivo.

#### 4.1.3. The Underlying Mechanism of Natural Active Ingredients Against Environmental Pollutant-Induced Reproductive Damage

As the toxicological mechanisms of environmental pollutants, such as microplastics and benzo[a]pyrene, on the male reproductive system become more comprehensively understood, research on the intervention of TCM compounds and natural active ingredients must transition from focusing on singular mechanisms to adopting a multidimensional and systematic approach. Current research primarily focuses on the toxicological impacts of individual pollutants, such as polystyrene microplastics and cadmium chloride. However, in real-world environments, pollutants frequently exist as complex mixtures, potentially intensifying reproductive toxicity through synergistic interactions. Future research should prioritize the development of animal models that replicate exposure to multiple pollutants simultaneously and systematically evaluate the comprehensive protective effects of TCM, such as YSTLF, in these complex exposure scenarios. Such research will provide an empirical basis for the clinical application of TCM in real-world environmental risk contexts.

Additionally, the potential of TCM compounds to safeguard intergenerational reproductive health merits further exploration. Studies on the ZGP have demonstrated its ability to enhance reproductive function across generations by alleviating testicular oxidative stress in male offspring exposed to prenatal stress. Building on this foundation, future research should investigate whether natural active compounds, such as resveratrol, can inhibit the transgenerational transmission of reproductive toxicity induced by environmental pollutants. This exploration should focus on elucidating the intervention pathways at the level of epigenetic regulatory mechanisms, including DNA methylation, histone modification, and non-coding RNA expression, thereby providing novel theoretical support for prevention and treatment of environmental reproductive hazards by TCM.

#### 4.1.4. Translational Application of TCM Active Ingredients in Enhancing Assisted Reproduction

In the field of assisted reproductive technology, research on the application of TCM compounds and natural active ingredients remains at a nascent stage. Future studies should focus on optimizing sperm cryoprotectants. Current evidence suggests that natural active ingredients, such as *Lycium barbarum* polysaccharide and rutin, can significantly improve sperm motility and DNA integrity following freezing and thawing. However, a standardized protocol for incorporating these TCM active ingredients into sperm cryoprotectants has not yet been established. Future research should consider the physiological characteristics of human sperm to systematically determine the optimal concentrations and freezing procedures, thereby developing a compound formulation system that integrates “natural ingredients with traditional cryoprotectants.” Furthermore, the in vitro fertilization model should be utilized to evaluate the effects of these active ingredients on embryo implantation rates and clinical pregnancy outcomes, ultimately providing a novel strategy to enhance the success rate of assisted reproductive technology.

### 4.2. Conclusions

This paper reviews the molecular mechanisms through which TCM compounds and natural active ingredients improve male reproductive function by regulating oxidative stress. The main conclusions are as follows:

1. TCM compounds exert protective effects on reproductive health via multi-target and multi-pathway mechanisms. Classical formulations such as WZYZP and BZBSP, based on the TCM principle of “tonifying the kidney and benefiting essence,” act synergistically to enhance antioxidant capacity, inhibit apoptosis, regulate reproductive endocrine hormones, and restore blood-testis barrier integrity. This highlights the holistic and multi-targeted regulatory advantages of TCM.
2. Natural active ingredients, including flavonoids, polyphenols, terpenes, alkaloids, and polysaccharides, share oxidative stress regulation as a common mechanism but differ in specific pathways. For instance, flavonoids and polyphenols primarily activate the Nrf2 pathway to upregulate antioxidant enzymes, whereas polysaccharides directly scavenge ROS and modulate mitochondrial function to protect reproductive health.
3. These findings provide a theoretical basis and potential drug development strategies for idiopathic male infertility treatment. TCM-derived compounds and natural ingredients show promise in mitigating oxidative stress-induced reproductive damage. Future research should focus on elucidating ingredient synergies, improving bioavailability, exploring their role against environmental pollutant-induced damage, and expanding applications in assisted reproductive technologies to bridge basic research and clinical practice.

## Figures and Tables

**Table 1:** Summary of classic TCM prescriptions for improving male reproductive function.

Category of TCM Compound	Representative Prescriptions	Most Important Bioactive Constituents	Tested Doses of TCM	Composition Medicinal Materials	Core Role Model	Regulation Effect of Oxidative Stress	Key Signaling Pathways/Molecular Targets	References
Nourish the kidneys and benefit essence	Wuzi Yanzong Prescription	Hyperoside, Quercetin, Rutin, Apigenin, Ferulic acid, Aucubin, Matrine, *Lycium barbarum* polysaccharide	Mouse (in vivo): 1 g/kg (gavage, daily, for 3 weeks); TM4 cell (in vitro): 0.2 mg/mL, 1 mg/mL, 5 mg/mL for 24 h	Lycii Fructus, Cuscutae Semen, Schisandrae Chinensis Fructus, Rubi Fructus, Plantaginis Semen	X-ray induced testicular injury in mice, H_2_O_2_ induced Sertoli cell (TM4) injury in mice	Decrease: MDA, OSI, Apoptosis rate, caspase-3 mRNA expression Increase: SOD activity, PCNA expression	Antioxidant enzyme system (SOD), PCNA	[29,30]
	Bazi Bushen Prescription	Hyperoside, Quercetin, Rutin, Icariin, Apigenin, Ferulic acid, Echinacoside, Ginsenoside Rg1, Aucubin, Epimedium alkaloids, Matrine, *Lycium barbarum* polysaccharide,	Mouse (in vivo): 0.7, 1.4, and 2.8 g/kg (gavage, daily, for 65 days)	Cuscutae Semen, Lycii Fructus, Schisandrae Chinensis Fructus, Rubi Fructus, Cnidii Fructus, Rosae Laevigatae Fructus, Semen Allii Tuberosi, Toosendan Fructus, Epimedii Herba, Morindae Officinalis Radix, Cistanches Herba, Rehmanniae Radix Praeparata, Cyathulae Radix, Ginseng Radix et Rhizoma, Cervi Cornu Pantotrichum, Hippocampus	Rapid aging mouse model induced by D-galactose/sodium nitrite	Decrease: MDA, 8-OH-dG Increase: TAC, GSH/GSSG ratio	Antioxidant enzyme system (TAC, GSH/GSSG)	[31]
	Zuogui Pill	Hyperoside, Quercetin, Rutin, Apigenin, Ferulic acid, Aucubin, Matrine, *Lycium barbarum* polysaccharide	Rats (in vivo): 1.89 g/kg (gavage, daily, for 21 days)	Rehmanniae Radix Praeparata, Cornus Fructus, Dioscoreae Rhizoma, Cervi Cornus Colla, Testudinis Carapacis et Plastri Colla, Lycii Fructus, Cuscutae Semen Achyranthis Bidentatae Radix	Reproductive injury model of male offspring induced by stress during pregnancy	Decrease: MDA, Bax, Cx43, Caspase-3 expression Increase: SOD activity	Apoptotic pathway (Bax/Caspase-3), Antioxidant enzyme system (SOD)	[32]
	Yishen Tongluo Formula	Quercetin, Rutin, Icariin, Ferulic acid, Aucubin, Epimedium alkaloids, Matrine, *Astragalus* polysaccharides	Rats (in vivo): 1.044 g/mL (1 mL/100 g body weight, gavage, for 8 weeks); Rats (in vivo):1.2 g/mL (1 mL/100 g body weight, gavage, for 30 days)	Cuscutae Semen, Rehmanniae Radix Praeparata, Epimedii Folium, Astragali Radix, Salviae Miltiorrhizae Radix et Rhizoma, Hirudo, Achyranthis Bidentatae Radix	Polystyrene microplastics/benzo[a]pyrene induced sperm DNA damage model	Decrease: DFI, MDA, NO Increase: SOD activity, ATP content	Antioxidant enzyme system (SOD), Regulation of energy metabolism (ATP)	[33,34]
	Qilin Pill	Hyperoside, Quercetin, Rutin, Icariin, Apigenin, Resveratrol, Ferulic acid, Epimedium alkaloids, Matrine, *Lycium barbarum* polysaccharide	Rats (in vivo): 1.62 g/kg, 3.24 g/kg (gavage, for 60 days)	Polygoni Multiflori Radix, Ecliptae Herba, Epimedii Folium, Cuscutae Semen, Cistanches Herba, Codonopsis Radix, Curcumae Radix, Lycii Fructus, Rubi Fructus, Dioscoreae Rhizoma, Salviae Miltiorrhizae Radix et Rhizoma, Astragali Radix, Paeoniae Radix Alba, Citri Reticulatae Pericarpium Viride, Mori Fructus	Oligoasthenospermia model induced by Tripterygium Glycosides	Decrease: ROS, MDA, Apoptosis related proteins (Bax, Cyt C, caspase-9/3) Increase: SOD activity, Tssk2 expression and reproductive hormone (FSH, LH, FT) levels	Mitochondrial apoptosis pathway (Bax-caspase-9), spermatogenesis related genes (TSSK2)	[35,36]
Warm and invigorate kidney Yang	Shenrong Pill	Quercetin, Rutin, Ferulic acid, Echinacoside, Ginsenoside Rg1, Aucubin, Matrine, *Lycium barbarum* polysaccharide, *Angelica* polysaccharide	TM3 cell (in vitro): Drug containing serum concentrations of 7.5%, 10%, and 12.5%	Ginseng Radix Rubra, Cervi Cornu Pantotrichum, Morindae Officinalis Radix, Cinnamomi Cortex, Cistanches Herba, Lycii Fructus, Cuscutae Semen, Rehmanniae Radix Praeparata, Poria, Astragali Radix, Paeoniae Radix Alba, Atractylodis Macrocephalae Rhizoma, Citri Reticulatae Pericarpium, Angelicae Sinensis Radix, Achyranthis Bidentatae Radix, Dioscoreae Rhizoma, Foeniculi Fructus, Glycyrrhizae Radix et Rhizoma	Oxidative damage of Leydig cells (TM3) induced by H202	Decrease: LPO, MDA Increase: SOD-1, CAT, GSH-Px activity	Antioxidant enzyme system (SOD-1/CAT/GSH-Px)	[37]
	Jinkui Shenqi Pill	Quercetin, Aucubin	Rats (in vivo): 0.5 g/mL (10 mL/kg, (gavage, daily, for 20 days); Mouse (in vivo): 1.2 g/kg (gavage, daily, for 35 days)	Rehmanniae Radix Praeparata, Dioscoreae Rhizoma, Cornus Fructus, Aconiti Lateralis Radix Praeparata, Cinnamomi Ramulus, Alismatis Rhizoma, Poria Moutan Cortex	Cyclophosphamide induced oligoasthenospermia and cortisone induced kidney Ying deficiency	Decrease: MDA Increase: SOD activity, testosterone level, Nrf2 pathway related gene expression	Nrf2 signaling pathway, Antioxidant enzyme system (SOD)	[38,39]
Benefit Qi and nourish blood-alongside	Bazhen Decoction	Quercetin, Rutin, Hesperetin, Ferulic acid, Ginsenoside Rg1, Aucubin, *Angelica* polysaccharide	Mouse (in vivo): 2.5 g/kg (gavage, daily, for 4 weeks)	Ginseng Radix et Rhizoma, Rehmanniae Radix Praeparata, Atractylodis Macrocephalae Rhizoma, Poria, Angelicae Sinensis Radix, Paeoniae Radix Alba, Chuanxiong Rhizoma, Glycyrrhizae Radix et Rhizoma	Decline of spermatogenic function in aged mice	Decrease: MDA Increase: SOD activity, testosterone level	Antioxidant enzyme system (SOD)	[40]
	Danggui Buxue Decoction	Quercetin, Apigenin, Ferulic acid, *Angelica* polysaccharide, *Astragalus* Polysaccharides	Rats (in vivo): 6 g/kg, 12 g/kg (gavage, daily, for 12 weeks)	Astragali Radix, Angelicae Sinensis Radix	Testicular injury induced by high fat diet	Decrease: MDA, apoptosis rate Increase: SOD activity, total sperm count, sperm motility	Antioxidant enzyme system (SOD), inhibition of apoptosis	[41]

**Table 2:** Molecular mechanism of natural active ingredients from TCM prescriptions regulating oxidative stress and improving male reproductive function.

Composition Category	Active Ingredient	Source of TCM Prescription	Tested Doses of Natural Active Ingredients from TCM	Core Role Model	Regulation Effect of Oxidative Stress	Key Signaling Pathways/Targets	References
Polyphenols	Resveratrol	Qilin Pill	Bull sperm (in vitro): 5, 10, 25, 50 µmol/L; Boar sperm (in vitro): 25, 50, 75, 100, 125, 150 µmol/L	Bull sperm oxidative stress model, boar sperm cryopreservation/liquid preservation model	Decrease: ROS, MDA, LPO Increase: SOD, CAT, GSH activity, AMPK phosphorylation, sperm motility, mitochondrial activity	AMPK pathway, antioxidant enzyme system	[74,75,76]
	Ferulic acid	Wuzi Yanzong Prescription, Bazi Bushen Prescription, Zuogui Pill, Qilin Pill, Shenrong Pill, Bazhen Decoction, Danggui Buxue Decoction, Yishen Tongluo Formula	Rats (in vivo): 50 mg/kg alternative day and 50 mg/kg daily (gavage, for 10 weeks); Rats (in vivo): 20 mg/kg (gavage, daily, for 7 days); Human sperm (in vitro): 0, 0.1, 0.2, 0.4, 0.8, 1.6 mmol/L	Diabetic rat testicular injury mode, cadmium induced testicular toxicity model, human sperm model	Decrease: ROS, MDA, NO Increase: SOD, CAT, GSH-Px activity, cAMP, cGMP, sperm motility, testosterone	Nrf2 signaling pathway, TGF-β1/Akt pathway, cyclic nucleotide signaling	[77,78,79]
	Echinacoside	Bazi Bushen Prescription, Shenrong Pill	TM3 cells (in vitro):: 50, 100, 200 µmol/L; 25, 50, Rats (in vivo): 100 mg/kg (gavage, daily, for 30 days)	Lead acetate induced testicular injury model, H_2_O_2_ induced TM3 cell injury model	Decrease: ROS, MDA, p-p38, p-JNK Increase: SOD, GSH, LDH activity, sperm motility	MAPK signaling pathway (p38/JNK)	[80]
Polyphenols (Flavonoids)	Hyperoside	Wuzi Yanzong Prescription, Bazi Bushen Prescription, Zuogui Pill, Qilin Pill	Mouse (in vivo): 12.5, 25, 50 mg/kg (gavage, daily, for 2 weeks); GC-2 cells (in vitro): 50, 100, 200 µmol/L; Human sperm (in vitro): 0, 5, 50, 100, 500 µmol/L	Triptolide induced testicular injury model, H_2_0_2_ induced GC-2 cell injury model and human sperm oxidative stress model	Decreas: ROS, MDA, LPO, DFI, FSH, LH Increase: SOD, GSH-Px, CAT activity, Nrf2 nuclear translocation, HO-1 expression	Keap1-Nr2-HO-1 signaling pathways	[81,82,83]
	Quercetin	Wuzi Yanzong Prescription, Bazi Bushen Prescription, Zuogui Pill, Yishen Tongluo Formula, Qilin Pill, Jinkui Shenqi Pill, Shenrong Pill, Bazhen Decoction, Danggui Buxue Decoction	Rats (in vivo): 20 mg/kg (gavage, daily, for 4 weeks); Human sperm (in vitro): 0, 0.025, 0.05, 0.1, 0.25, 0.5, 10, 50 µmol/L	Cadmium chloride induced testicular toxicity transverse type, leukocytospermia model, human sperm cryopreservation transverse type	Decreas: ROS, MDA, H_2_O_2_, DFI Increase: SOD, CAT, GSH-Px activity, TAC, sperm motility, Cytb, NADH5	Antioxidant enzyme system (SOD/CAT/GSH-Px)	[84,85,86,87]
	Rutin	Wuzi Yanzong Prescription, Bazi Bushen Prescription, Zuogui Pill, Yishen Tongluo Formula, Qilin Pill, Shenrong Pill, Bazhen Decoction	Boar sperm (in vitro): 0.2, 0.4, 0.6, 1.0, 2.0 mmol/L; Rams epididymal sperm (in vitro): 0.5, 0.75, 1.0, 1.25 mmol/L	Boar/ram sperm cryopreservation model	Decrease: ROS, MDA Increase: SOD, CAT, GSH-Px activity, sperm motility, mitochondrial activity, acrosome integrity	Antioxidant enzyme system (SOD/CAT/GSH-Px)	[77,78]
	Icariin	Bazi Bushen Prescription, Yishen Tongluo Formula, Qilin Pill	Mouse (in vivo): 40 mg/kg, 80 mg/kg (gavage, daily, for 12 weeks); Leydig cell (in vitro): 0.2 µg/mL, 1 µg/mL, 5 µg/mL (pre-treatment for 3 h); Human sperm (in vitro): 0.001–0.100 µg/mL	DEHP induced Leydig cell injury model, human sperm oxidative stress model	Decrease: ROS, MDA, Nuclear translocation of NF- κB p65 Increase: SOD activity, AMPK phosphorylation, Nrf2, testosterone, sperm motility, LDH	Ampk-Nrf2 pathway, NF-κB p65	[54,88,89]
	Hesperetin	Bazhen Decoction	Rats (in vivo): 50 mg/kg (gavage, daily, for 46 days); Human sperm (in vitro): 20 µmol/L	Diabetic rat testicular injury model, human sperm cryopreservation model	Decrease: ROS, MDA, DFI, caspase-3, inflammatory factors Increase: SOD, GSH, CAT, GSH-Px activity, sperm survival rate and normal morphology rate	Antioxidant defense system, anti-inflammatory pathway	[79,90]
	Apigenin	Wuzi Yanzong Prescription, Bazi Bushen Prescription, Zuogui Pill, Qilin Pill, Danggui Buxue Decoction	Rats (in vivo): 234 mg/kg, 468 mg/kg (gavage, conducted 6 days per week for 12 weeks; 117 mg/kg, 234 mg/kg, 35 mg/kg (gavage, conducted 6 days per week for 4 weeks) Bull sperm (in vitro): 0.2, 0.4, 0.6, 0.8 mmol/L	Acrylonitrile induced sperm damage model, bull sperm cryopreservation model	Decrease: ROS, MDA, DFI, ASK1, p-JNK/p38 Increase: SOD, GSH-Px, CAT activity, sperm motility and acrosome integrity	ASK1-JNK/p38 pathway, antioxidant enzyme system	[91,92,93]
Terpenoids	Ginsenoside Rg1	Bazi Bushen Prescription, Shenrong Pill, Bazhen Decoction	Mouse (in vivo): 20 mg/kg (injected intraperitoneally for 28 days); Mouse (in vivo): 40 mg/kg (injected intraperitoneally for 27 days)	Aging testis model induced by D-galactose	Decrease: MDA, P53, P21 Increase: SOD activity, TAC, Nrf2, HO-1, NQO1, GCLC, GCLM expression	Nrf2/HO-1 signaling pathway, aging related pathways (P53/P21)	[94,95,96]
	Aucubin	Wuzi Yanzong Prescription, Bazi Bushen Prescription, Zuogui Pill, Yishen Tongluo Formula, Shenrong Pill, Jinkui Shenqi Pill, Bazhen Decoction.	Mouse (in vivo): 5, 10, 20 mg/kg (injected intraperitoneally, daily, for 2 weeks); TM4 cells (in vitro): 2, 5, 10, 20, 40 µmol/L	Tripterygium wilfordii induced testicular injury model, H_2_O_2_ induced TM4 cell injury model	Decrease: ROS, MDA, JNK phosphorylation Increase: SOD, GSH activity, Nrf2 nuclear translocation, NQO1, HO-1 expression	Nrf2-NQO1/HO-1 pathway, ROS/JNK pathway	[97,98]
Alkaloids	*Epimedium* alkaloids	Bazi Bushen Prescription, Yishen Tongluo Formula, Qilin Pill	Mouse (in vivo): 50, 100, 200 mg/kg (gavage, daily, for 30 days)	Cyclophosphamide induced reproductive system damage model	Decrease: MDA, Bax Increase: SOD activity, testosterone, Bcl-2, Bcl-2/Bax ratio	Antioxidant enzyme system (SOD), apoptosis pathway (Bcl-2/Bax)	[99]
	Matrine	Wuzi Yanzong Prescription, Bazi Bushen Prescription, Zuogui Pill, Yishen Tongluo Formula, Qilin Pill, Shenrong Pill	Rats (in vivo): 100 mg/kg (injected intraperitoneally, daily, for 4 weeks)	Model of testicular toxicity induced by permethrin	Decrease: ROS, MDA, p-ERK1/2, COX-2 Increase: SOD activity, StAR gene expression, testosterone, sperm count	ERK/COX-2 pathway, steroids synthesis regulation (StAR)	[100]
Polysaccharides	*Lycium barbarum* polysaccharide	Wuzi Yanzong Prescription, Bazi Bushen Prescription, Zuogui Pill, Qilin Pill, Shenrong Pill	Mouse (in vivo): 10, 33.3, 100 mg/kg (gavage, daily, for 35 days); Mouse (in vivo): 0.2, 0.4, 0.6 g/kg (gavage, daily, for 5 days); Human sperm (in vitro): 1000 µg/mL	Cadmium induced oligoasthenospermia model, cyclophosphamide induced reproductive toxicity model, cryopreservation model of human sperm	Decrease: ROS, MDA, NO, DFI, Bax Increase: SOD, GSH-Px activity, Bcl-2, sperm motility and mitochondrial membrane potential	Regulation of antioxidant enzyme system and mitochondrial function	[101,102,103]
	*Angelica* polysaccharide	Shenrong Pill, Bazhen Decoction, Danggui Buxue Decoction	Mouse (in vivo): 140 mg/kg (injected intraperitoneally, daily, for 27 days); Sheep sperm (in vitro): 200, 400, 1000, 2000 mg/L; Goat sperm (in vitro): 600 µg/mL	D-galactose-induced aging testis model, sheep/goat sperm cryopreservation model	Decrease: MDA, P53, P21 Increase: SOD activity, TAC, sperm motility and acrosome integrity, CAT	Antioxidant defense system, aging related pathways (P53/P21)	[104,105,106]
	*Astragalus* polysaccharides	Yishen Tongluo Formula, Danggui Buxue Decoction.	Mouse (in vivo): 100 mg/kg (gavage, daily, for 14 days); 200 mg/kg (gavage, daily, for 8 weeks); Boar sperm (in vitro): 0.25, 0.5, 0.75, 1 mg/mL; Bovine sperm (in vitro): 0.2, 0.3, 0.5 mg/mL	Cantharidin induced testicular injury model, DBP induced reproductive toxicity model, boar/bovine sperm preservation model	Decrease: ROS, MDA, Beclin-1, LC3-II Increase: SOD, CAT, GSH-Px activity, mTOR, P-PKAs, ATP, sperm motility	Nrf2-Keap1 pathway, PI3K/AKT/mTOR pathway, cAMP-PKA pathway	[107,108,109,110]

## Data Availability

No new data were created or analyzed in this study.

## Abbreviations

The following abbreviations are used in this manuscript:

AMP	Adenosine 5′-monophosphate
AMPK	AMP-activated protein kinase
ATP	Adenosine triphosphate
AKT	Protein kinase B
ARE	AU-rich element
BZBSP	Bazi Bushen Prescription
Bcl-2	B-cell lymphoma 2
Bax	BCL-2 associated X protein
BZD	Bazhen Decoction
CAT	Catalase
Cyt b	Cytochrome b
Cyt c	Cytochrome c
cAMP	cyclic adenosine monophosphate
cGMP	cyclic guanosine monophosphate
CdCl_2_	cadmium chloride
COX-2	Cyclooxygenase 2
DBD	Danggui Buxue Decoction
D-gal	D-galactose
DFI	DNA fragmentation index
DBP	Dibutyl phthalate
DEHP	Di (2-ethylhexyl) phthalate
FT	Free testosterone
FSH	Follicle-stimulating hormone
FeAA	ferrous ascorbate
GSH	Glutathione
GSSG	oxidized glutathione
GSH-Px	Glutathione peroxidase
GnRH	Gonadotropin-releasing hormone
H_2_O_2_	hydrogen peroxide
HO-1	Heme oxygenase-1
JKSQP	Jinkui Shenqi Pill
JNK	c-Jun N-terminal kinase
Keap1	Kelch-like ECH-associated protein 1
LH	Luteinizing hormone
LPO	Lipid peroxidation products
MDA	Malondialdehyde
MMP	Mitochondrial membrane potential
MAPK	Mitogen-activated protein kinase
NO	Nitric oxide
Nrf2	Nuclear factor E2-related factor 2
NF-κB p65	Nuclear factor kappa B p65
NQO1	Quinone oxidoreductase 1
OSI	Oxidative stress index
PCNA	Proliferating cell nuclear antigen
p38	p38 mitogen-activated protein kinase
p-p38	Phosphorylated p38
p-JNK	Phosphorylated JNK
p-ERK1/2	Phosphorylated extracellular signal-regulated kinase 1/2
PKA	Protein kinase A
p-PKAs	Protein kinase A substrate phosphorylation
QLP	Qilin Pill
ROS	Reactive oxygen species
SRP	Shenrong Pill
SOD	Superoxide dismutase
SOD-1	Superoxide dismutase-1
STZ	Streptozotocin
StAR	Steroidogenic acute regulatory protein
TCM	Traditional Chinese Medicine
TAC	total antioxidant capacity
TP	Triptolide
TSSK2	Testicular-specific serine kinase 2
WZYZP	Wuzi Yanzong Prescription
YSTLF	Yishen Tongluo Formula
ZGP	Zuogui Pill
8-OH-dG	8-hydroxydeoxyguanosine
